# Revisiting the expression and function of follicle-stimulation hormone receptor in human umbilical vein endothelial cells

**DOI:** 10.1038/srep37095

**Published:** 2016-11-16

**Authors:** Joanna Stelmaszewska, Marcin Chrusciel, Milena Doroszko, Malin Akerfelt, Donata Ponikwicka-Tyszko, Matthias Nees, Marco Frentsch, Xiangdong Li, Jukka Kero, Ilpo Huhtaniemi, Slawomir Wolczynski, Nafis A. Rahman

**Affiliations:** 1 Department of Reproduction and Gynecological Endocrinology, Medical University of Bialystok, 15276 Bialystok, Poland; 2 Institute of Biomedicine, Department of Physiology, University of Turku, 20520 Turku, Finland; 3 Department of Biology and Pathology of Human Reproduction, Institute of Animal Reproduction and Food Research, Polish Academy of Science, 10714 Olsztyn, Poland; 4 Institute of Biomedicine, Department of Cell Biology and Anatomy, University of Turku, 20520 Turku, Finland; 5 Regenerative Immunology and Aging, Berlin-Brandenburg Center for Regenerative Therapies, CVK Charité University Medicine, 13353 Berlin, Germany; 6 State Key Lab for Agrobiotechnology, China Agriculture University, 100193 Beijing, China; 7 Institute of Reproductive and Developmental Biology, Imperial College London, W12 ONN London, UK

## Abstract

Expression of follicle-stimulation hormone receptor (*FSHR*) is confined to gonads and at low levels to some extragonadal tissues like human umbilical vein endothelial cells (HUVEC). FSH-FSHR signaling was shown to promote HUVEC angiogenesis and thereafter suggested to have an influential role in pregnancy. We revisited hereby the expression and functionality of FSHR in HUVECs angiogenesis, and were unable to reproduce the FSHR expression in human umbilical cord, HUVECs or immortalized HUVECs (HUV-ST). Positive controls as granulosa cells and HEK293 cells stably transfected with human FSHR cDNA expressed FSHR signal. In contrast to positive control VEGF, FSH treatment showed no effects on tube formation, nitric oxide production, wound healing or cell proliferation in HUVEC/HUV-ST. Thus, it remains open whether the FSH-FSHR activation has a direct regulatory role in the angiogenesis of HUVECs.

Follicle-stimulating hormone (FSH) is a heterodimeric glycoprotein hormone produced in the anterior pituitary gland. FSH binds to its receptor (FSHR, a G-protein coupled receptor) localized mainly in ovarian granulosa and testicular Sertoli cells^1^. FSH-FSHR activation is crucial for the fertility of females^2^ and males^3^. Among the physiological functions of FSH, in the female it promotes the maturation of ovarian follicles, stimulates aromatase expression and the secretion of estrogens. In the male, FSH regulates indirectly spermatogenesis by stimulating the metabolism of Sertoli cells^4^,^5^. FSH binding to the extracellular domain of FSHR activates mainly the Gαs/cAMP/PKA signalling pathway^6^, which has been considered as the canonical signal transduction pathway for FSHR. In addition, the Gαs-independent pathways, such us the PI3K/PIP3–AKT/mTOR pathway, β-arrestin-dependent pathway or interaction of FSHR with PPL1, FoxO1a and 14-3-3τ, are involved in FSH-dependent cellular processes [reviewed in ref. 7].

Recent reports on FSHR expression in extragonadal tissues have opened up potentially novel aspects of its function. FSHR has been shown to be expressed in human endometrium^8^, myometrium^8^, placenta^8^, fallopian tube^8^, endometriosis lesions^9^, and also in the vascular endothelium of different types of tumors (prostate, breast, colon, pancreas, urinary bladder, kidney, lung, liver, stomach, testis, and ovary)^10^,^11^,^12^, including their metastases^13^. With regard to tumor vessel cells (primary and metastatic), it was suggested that FSHR might serve as a potential cellular marker of different tumors and provide a novel approach for targeted cancer therapy. Four-years after the report of FSHR expression in tumor vessel cells^14^, FSHR expression was demonstrated in human umbilical vein endothelial cells (HUVEC)^15^. HUVECs significantly responded to FSH treatment in a series of functional tests like tube formation, wound healing, cell migration and proliferation, nitric oxide production and cell survival^15^. This allowed the authors of above study to propose that HUVECs express functional FSHR expression and that the FSH-FSHR activation promotes angiogenesis as effectively as the potent well-characterized angiogenic factor VEGF^15^. Based on these functional data they further suggested that FSH-FSHR could have an influential role in placental development and healthy pregnancy^15^. HUVECs have served widely as a model for studying placental angiogenesis *in vitro*^16^. This is contradictory to the observation that both males and females with inactivating *FSHR* mutations, thus obligatorily devoid of functional FSHR in their placenta, develop normally in utero^17^.

Due to these recent findings on functional FSHR expression in HUVECs and FSH-FSHR activation of angiogenesis, we revisited the issue of FSHR expression and function in HUVECs, along with an array of convincing positive and negative controls.

## Results

### FSHRs are not expressed in HUVECs

**Figure 1:**
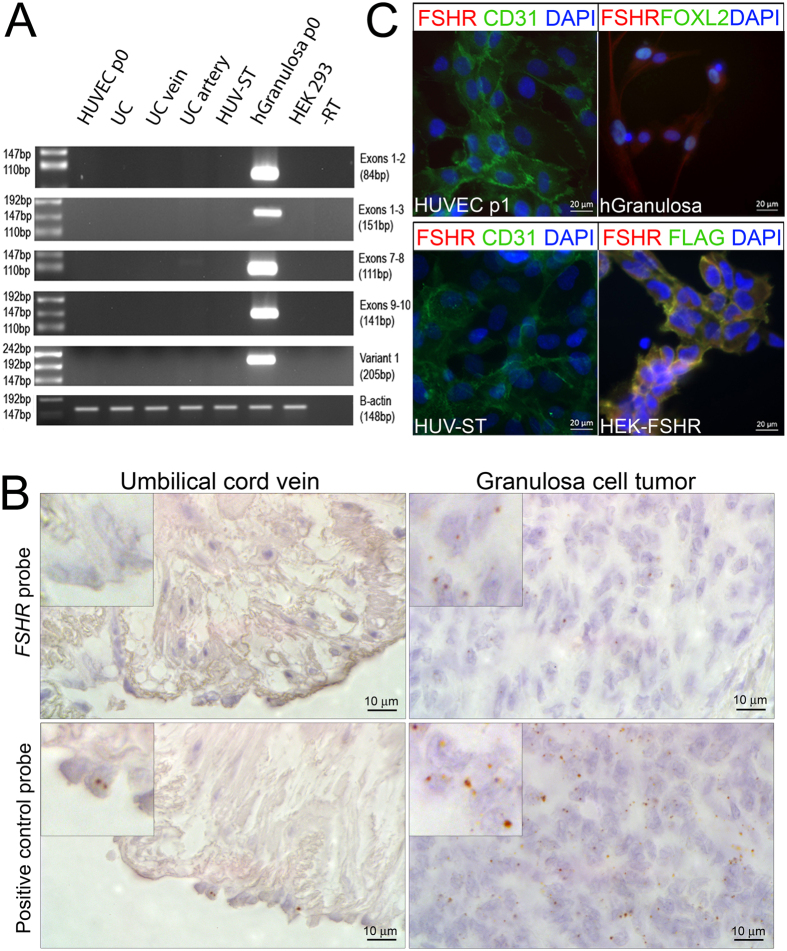
Expression and localization of FSHR in human umbilical vein endothelial cells (HUVEC). (**A**) *FSHR* expression was analysed with primers spanned different exons of *FSHR* using cDNA from primary HUVECs (passage 0), umbilical cord (UC), umbilical cord vein (UC vein), umbilical cord artery (UC artery), HUV-ST (SV40Tag/telomerase-immortalized human umbilical vein endothelial cell line) cell line and human granulosa cells passage 1, as a positive control. A no-reverse transcriptase control (-RT) and no template control (H_2_O) were used as negative controls. Beta-actin (B-actin, *ACTB*) was used as housekeeping gene. A representative picture of cropped gel has been shown here, full-length blots/gels are presented in Supplementary information file. (**B**) *RNAScope In situ* hybridization of *FSHR* was performed in umbilical cord and granulosa cell tumour formalin fixed paraffin embedded sections. *POLR2A (lower panel*), a positive control probe for low expression was used as a sections’ quality control. (**C**) Immunocytochemical co-localization of FSHR and CD31 in HUVECs and HUV-ST cells. Human granulosa cells and HEK293 cells stably transfected with human FSHR cDNA (HEK-FSHR cells) were used as FSHR-positive control cells. FOXL2 was used as a granulosa cell marker where FLAG peptide was used as a reporter sequence to detect FSHR construct in HEK-FSHR cells. DAPI was used as counterstaining to detect cell nuclei (blue).

We determined the purity of the isolated endothelial cells by analyzing the expression of endothelial cell markers von Willebrand Factor and CD31 (Supplemental Fig. 1). In order to analyze the *FSHR* splice variants^18^,^19^, we carried out reverse transcription using two cDNA synthesis kits, SYBR-, and TaqMan-based qPCR analyses. Both gave identical results by not detecting transcription variants of *FSHR* in UC, vein, artery, HUVEC (passage 0) and/or HUV-ST cells (passage 3) (Fig. 1A, Supplemental Fig. 2). Human granulosa cells used as positive control showed *FSHR* exon-specific amplification bands, which we further confirmed by sequencing. FSHR RNAscope *in situ* hybridization analysis confirmed the negative and positive qPCR results (Fig. 1B). The *FSHR* specific probe showed no signal in UC vein (Fig. 1B). The human granulosa cell tumor sections used as positive control showed *FSHR* transcript localization in carcinoma cells (higher magnification inserts, Fig. 1B). Quality of UC sections and specificity of the RNA *in situ* hybridization assay was confirmed by the results of *POLR2A* probing (positive control) applied as a positive low-abundance control probe (Fig. 1B).

Despite the negative results of *FSHR* expression, we analyzed FSHR at protein level using the same mouse monoclonal FSHR-323 antibody as used in the earlier study^15^. Immunocytochemistry showed specific membrane and cytoplasmic staining for FSHR in both positive controls, i.e. in human granulosa cells that expressed FOXL2 and in HEK-293/FSHR cells, where HEK293 cells were stably transfected with human FSHR cDNA fused with FLAG peptide (Fig. 1C, Supplemental Fig. 3). No FSHR signal could be observed in HUVEC, HUV-ST or in wild type HEK-293 cells used as negative control (Fig. 1C, Supplemental Fig. 3). We used CD31, displaying membrane localization, as a positive marker for endothelial cells (Fig. 1C).

### FSH-FSHR activation does not influence the endothelial proangiogenic mechanisms

**Figure 2:**
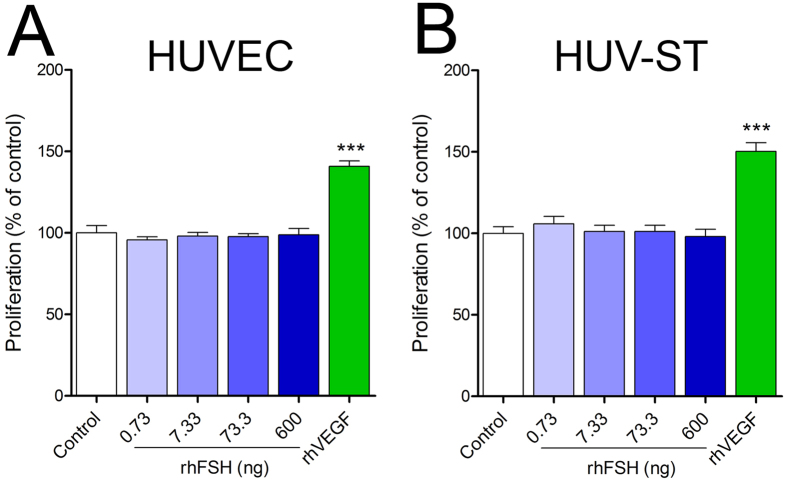
Proliferation of primary HUVECs (**A**) and HUV-ST (**B**) cells. Cells were stimulated without or with 0.733, 7.33, 73.3 and 600 ng/ml of rhFSH or 50 ng/ml of rhVEGF used as a positive control. Each bar represents the mean ± SEM of three independent experiments with n = 8 per treatment. Asterisks indicate differences between control and stimulated cells (***P < 0.001).

A direct proangiogenic signaling through FSH-FSHR on HUVECs was previously reported^15^. Despite the absence of FSHR at mRNA and protein levels, we further tried to reproduce the earlier functional experiments. We could not observe any rhFSH-stimulated increased proliferation in HUVEC (Fig. 2A) and/or HUV-ST cells (Fig. 2B) *vs*. control. Statistically significant difference was observed in proliferation rate only after rhVEGF stimulation used as a positive control (Fig. 2A,B).

**Figure 3:**
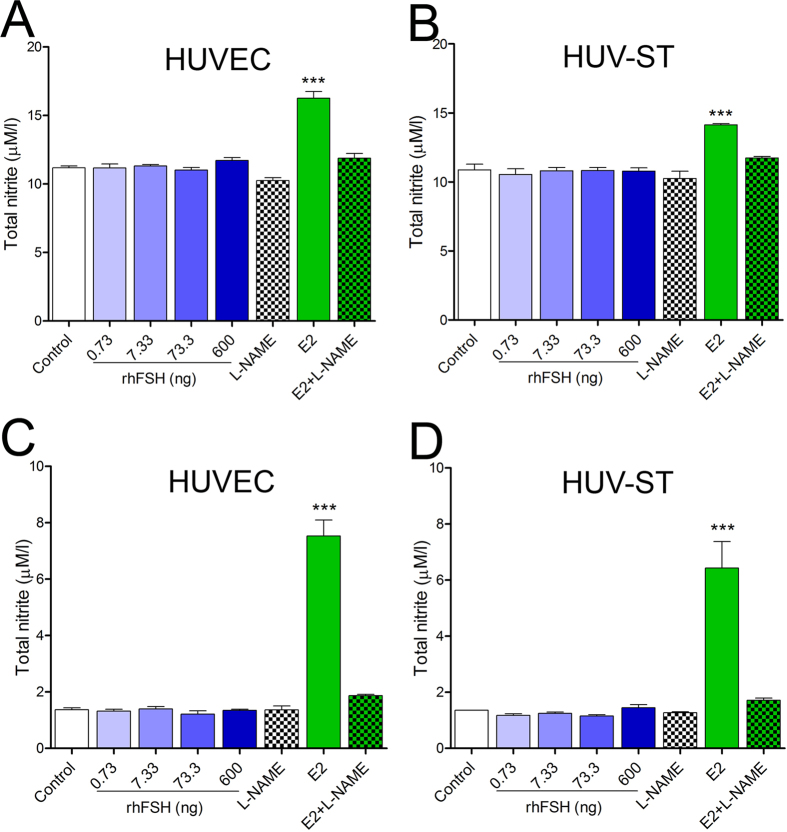
Production of NO in HUVECs (**A**,**C**) and HUV-ST (**B**,**D**). Cells were stimulated without or with 0.733, 7.33, 73.3 and 600 ng/ml of rhFSH for 4 h in normal basal medium (**A**,**B**) or for 1 h in HBSS (**C**,**D**). Estradiol (E2) was used as a positive control. NO production was inhibited by L-NG-Nitroarginine Methyl Ester (L-NAME). Each bar represents the mean ± SEM of three independent experiments with n = 4 per treatment. Asterisks indicate differences between control and stimulated cells (*P < 0.05, **P < 0.01, ***P < 0.001).

We then determined NO production in rhFSH-stimulated HUVEC and HUV-ST cells by measuring total nitrite in two different experimental setups for starving the cells: keeping them in either basal culture medium for 4 h (Fig. 3A,B) or in HBSS medium for 1 h (Fig. 3C,D). HUVEC and HUV-ST cell stimulation in HBSS was conducted in order to avoid nitrite/nitrate contamination from the medium/serum and protein interactions with Griess reagents (sulfanilamide and *N*-1-napthylethylenediamine) in Griess reaction^20^. A dose dependent treatment with rhFSH was unable to stimulate the NO production in HUVEC or HUV-ST cells (Fig. 3). Nitrite concentration was increased only after E2 stimulation (positive control)^21^. In both HUVEC and HUV-ST cells, NO synthase inhibitor L-NAME significantly decreased E2-stimulated nitrate production towards control/basal nitrite level in both cell types (Fig. 3A–D).

**Figure 4:**
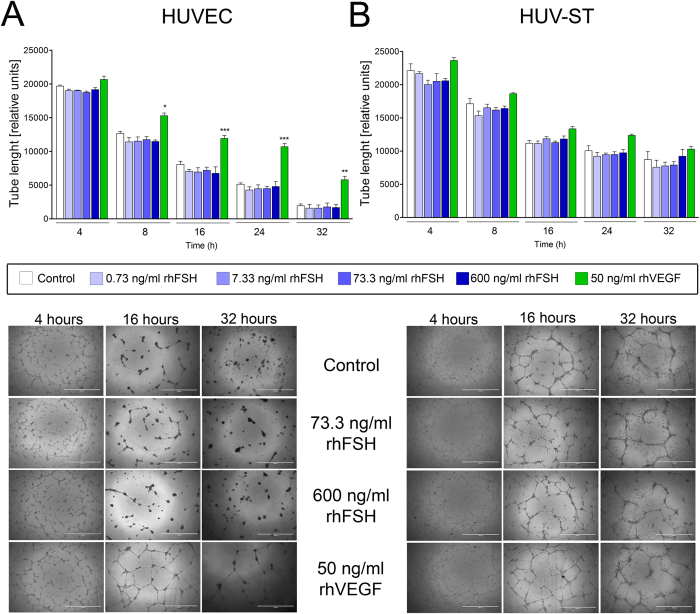
Tube formation of HUVECs (**A**) and HUV-ST (**B**) cells. Cells seeded on reduced growth factor basement membrane matrix were stimulated without or with 0.733, 7.33, 73.3 and 600 ng/ml of rhFSH, or 50 ng/ml of rhVEGF used as a positive control. Pictures taken after 4, 8, 16, 28 and 32 h and total tube length was measured by Angiogenesis Analyzer for ImageJ software and selectively confirmed manually. Each bar represents the mean ± SEM of three independent experiments with n = 5 per treatment. Asterisks indicate differences between control and stimulated cells (*P < 0.05, **P < 0.01, ***P < 0.001).

**Figure 5:**
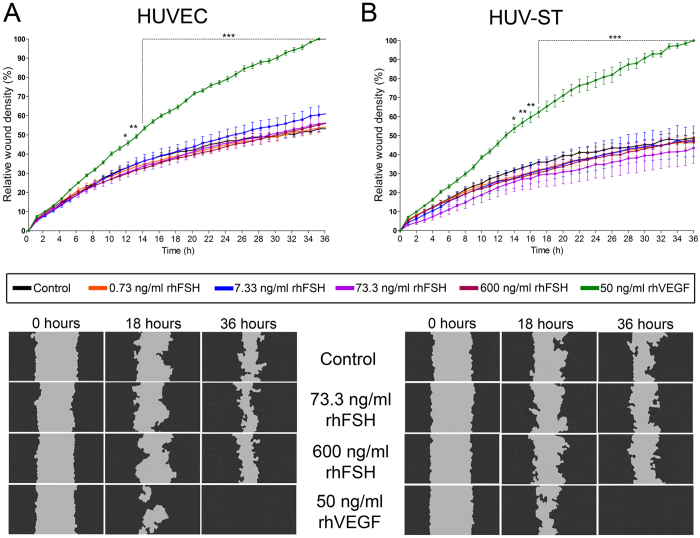
Cell migration measured by wound healing assay in HUVECs (**A**) and HUV-ST (**B**) cells. Cells were stimulated without or with 0.733, 7.33, 73.3 and 600 ng/ml of rhFSH, or 50 ng/ml of rhVEGF used as a positive control. *Upper panel* shows the relative wound density/time and *lower panel* shows the cell migration. Pictures were taken every hour by IncuCyte ZOOM^®^. Relative wound density was calculated by IncuCyte™ Chemotaxis Cell Migration Software. Each bar represents the mean ± SEM of three independent experiments with n = 6 per treatment/experiment. Asterisks indicate differences between control and stimulated cells (*P < 0.05, **P < 0.01, ***P < 0.001).

Next, using tube formation assay we analyzed the ability of rhFSH and VEGF-stimulated HUVEC and HUV-ST cells to form capillary-like network structures on a Matrigel basement membrane matrix. Tube formation by HUVEC and HUV-ST cells in response to a graded concentration of rhFSH was assessed after incubations for 4, 8, 16, 24 and 32 h (Fig. 4A,B; Supplemental Fig. 4A,B). rhFSH did not stimulate the tube formation in HUVEC or HUV-ST at any of the doses or at any time point *vs*. control (Fig. 4A,B; Supplemental Fig. 4A,B). Quantification of total tube lengths (from 8^th^ h of the experiment and onwards) and number of master segments (from 16^th^ h of the experiment) were significantly higher in rhVEGF-stimulated HUVEC cells *vs*. control (Fig. 4A; Supplemental Fig. 4A). rhFSH or rhVEGF stimulation had no effects on total tube length or number of master segments in HUV-ST cells (Fig. 4B; Supplemental Fig. 4B). There was a time-course dependent progressive decrease in total tube lengths and number of master segments in both cell types in all groups. The lower panel of Fig. 4 showed specimen pictures of tube formation at different selected time points and under rhFSH and rhVEGF stimulation. Another test used to study angiogenesis was wound healing assay, which measures the ability of cells to initiate migration and proliferation once a denuded area is created in a confluent culture^22^. rhFSH stimulation had no effects on endothelial cell migration/wound healing in both HUVEC and HUV-ST cells *vs*. control (Fig. 5A,B). Stimulation with rhVEGF resulted with a significant increase in cell migration in both HUVEC and HUV-ST cells from 12^th^ and 14^th^ h and onwards, respectively.

**Figure 6:**
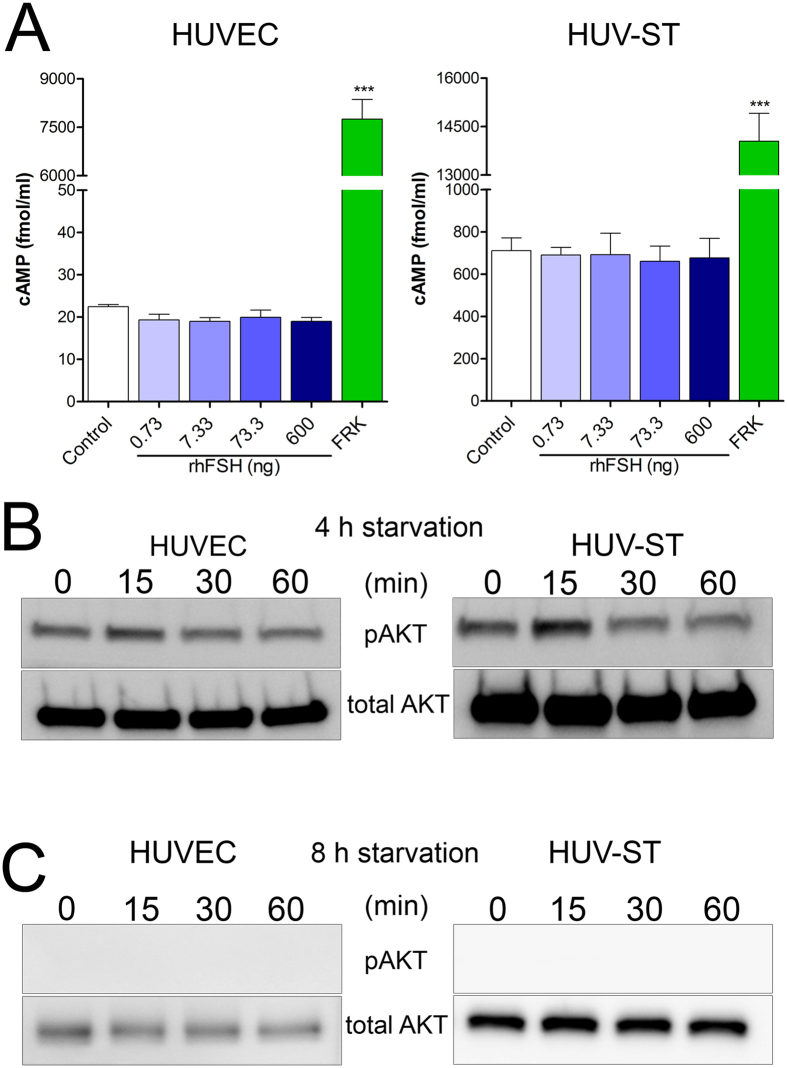
Production of total cAMP (**A**) and phosphorylation of AKT (**B**) in HUVECs and HUV-ST cells. cAMP was measured in medium collected from HUVEC and HUV-ST cells after 1 h incubation without or with 0.733, 7.33, 73.3, and 600 ng/ml of rhFSH, or 10 μM forskolin used as a positive control. Each bar represents the mean ± SEM of three independent experiments with n = 4 per treatment. Asterisks indicate differences between control and stimulated cells (***P < 0.001). *Middle* (**B**) *and Lower* (**C**) *panels* present the phosphorylation of AKT (pAKT) in HUVECs and HUV-ST cells. Cells starved for either 4 (**B**) or 8 h (**C**) were stimulated with 600 ng/ml of rhFSH for 15, 30 and 60 minutes. A representative picture of cropped gel has been shown here, full-length blots/gels are presented in Supplementary information file.

In contrast to forskolin, the rhFSH dose response stimulation of HUVEC and HUV-ST cells did not affect/stimulate cAMP production *vs*. control (Fig. 6A). No quiescence of Akt phosphorylation (pAkt) could be observed (no bands) in non-stimulated cells at time point “0” after 4 h of starvation, in either HUVEC or HUV-ST cells (Fig. 6B) in Western blot analysis, which is mandatory in order to analyze further the stimulatory effects. Starvation of HUVEC and HUV-ST cells for 8 h silenced basal phosphorylation (time point 0) of AKT and no AKT phosphorylation (pAkt) signal could not be observed after stimulation with 600 ng/mL of rhFSH at any time point (Fig. 6C).

## Disscusion

Extragonadal LHCGR and FSHR expression and their functionality have been of great interest in the recent years with lots of expectations of novel functions associated with them^9^,^10^,^11^,^23^,^24^,^25^,^26^,^27^,^28^,^29^. Especially the FSHR expression in vascular epithelium in a wide range of human tumors^11^,^14^ and their metastases^13^ pointed out the potential use of FSHR expression as a tumor marker or as a new target for cancer therapy. Another important finding was the novel functional expression of FSHR in HUVECs^15^, followed by further expression in extragonadal reproductive tissues in mice^8^. These findings opened up a novel role for FSH-FSHR signaling in angiogenesis and in broader sense in female reproductive physiology and pregnancy. In this study, we failed to reproduce the earlier results^15^ that FSHR is expressed in the endothelial cells of umbilical vein and drives proangiogenic functions through FSH-FSHR signaling in HUVECs.

In our study, we analyzed *FSHR* expression with two kits for reverse transcription and qPCR systems, both giving negative results for the HUVEC and HUV-ST cells. The PCR products from the positive control samples were sequenced in order to reconfirm their fidelity. Negative *FSHR in situ* hybridization additionally strengthened our qPCR results. The RNAscope *in situ* hybridization technology allows for single-transcript visualization with efficient background suppression^30^. *FSHR* mRNA was localized in granulosa cell tumor controls, but not in umbilical cord vein. As expected, we did not either detect FSHR at protein levels in HUVEC or HUV-ST cells. Representative agarose gels, immunocyto-, and immunohistochemistry pictures presented in the former study showed a discrepancy between FSHR mRNA and protein expression findings^15^. The exon specific amplification products indicated a traceable *FSHR* mRNA expression in HUVECs. However, in the umbilical cord immunohistochemistry, FSHR was localized in the endothelial cells of umbilical vein and smooth muscle cells of tunica media^15^. The representative figure of non-human primate ovary showed FSHR immunoreaction in granulosa cells, as well as in stromal and endothelial cells, which was not mentioned in the results or discussed^15^. Thus, one cannot rule out suboptimal immunohistochemical staining conditions or that the antibody lot used (produced and purified from FSHR-323 hybridoma cells) was not specific enough in this study. The majority of the FSHR reports in the extragonadal tissues were based mainly on the immunohistochemistry results using the *“in-house”* FSHR-323 antibody, usually without additional methodological confirmations^9^,^11^,^12^,^13^,^14^,^15^. This FSHR 323 antibody is not commercially available, and thus could not be independently validated.

In contrast to the former study^15^, we did not find any rhFSH stimulated positive effects on HUVEC and HUV-ST cells proliferation. Considering that HUVECs divide approximately every 18–24 hours^31^, the 2 and 4 h of stimulation time used in in the former study seems to be too short to observe marked changes in cell division between control and stimulated cells. In previous reports, the proliferation of HUVECs has been analyzed at least after 24 h incubation with stimulants^31^,^32^,^33^,^34^,^35^,^36^. This minimum coupling time allows the cells to complete their division cycle and sufficiently increase measurable amounts of newly synthetized DNA.

In the former study, the authors found only a very high dose of 600 ng/mL rhFSH (i.e. 8185.54 IU/L) could cause the proangiogenic effects in HUVECs^15^. Usually a maximum dose of 100 ng/mL^37^ or lower^38^,^39^ of rhFSH is able to cause the cAMP response in granulosa cells. Much higher (2–3-fold) concentrations are needed to stimulate inositol phosphate production^40^ in granulosa or rat Sertoli cells^41^. While osteoclasts were stimulated even with a concentration of 300 ng/mL^23^ or rat primary cells with 450 ng/mL^41^, no FSHR saturation was observed. We think the dose of 600 ng/mL rhFSH was too high to be physiologically meaningful, for which reason we stimulated the HUVECs and HUV-ST also at lower rhFSH concentrations.

HUVECs are not the best *in vitro* model to study NO formation^42^. Along with passaging (keeping for longer periods) these cells lose the expression of endothelial nitric oxide synthase (eNOS) and consequently NO formation decreases significantly^43^. We also observed a very low basal total nitrite concentration suggesting low NO formation even by early passages 1 and 2 of primary HUVECs. Additionally, the lack of changes in NO production after L-NAME constitutive NO synthase inhibition further suggested their low basal levels^21^,^44^. Both lines positively responded to estradiol stimulation, used often as a positive control^21^. Moreover, around 6-fold lower total nitrite concentration observed in HUVEC and HUV-ST cells stimulated in HBSS for 1 h in comparison to basal culture medium for 4 h may suggest nitrite/nitrate contamination of the latter. Although the same assay was used to measure total nitrite in the former study, the concentrations obtained in their experiment were below the kit’s assay range (3.12–200 μmol/L) and even for the mean minimum detectable dose (0.25 μmol/L), which may have affected the quantitative reliability of their results^15^. They stimulated HUVECs in T75 flasks and concentrated collected medium 5-fold using 10,000 molecular weight cut-off centrifugal filters before assay procedures. These 10,000 molecular weight cut-off centrifugal filters are recommended for samples deproteinization, not for samples concentration. Proteins are known to interfere with the Griess reaction, which was used in the applied assay to measure total nitrite^45^,^46^.

The tube formation assay is one of the most common assays to demonstrate the angiogenic activity of vascular endothelial cells *in vitro*^31^,^47^. This assay involves endothelial cell adhesion, migration, protease activity and tubule formation. The endothelial cells form capillary-like structures on basement membrane matrix and this process is accelerated or inhibited in response to proangiogenic or antiangiogenic signals found in conditioned media, respectively^31^,^47^. The tube formation by endothelial cells occurs quickly within 1–2 h and lumen-containing tubules within 2–3 h^47^. In contrast to the previous study^15^, we did not observe any significant effects on tube formation in HUVEC and HUV-ST cells after 4 h of rhFSH stimulation *vs*. control. On the contrary, our control non-stimulated cells were able to form capillary like structures with full lumens. This was in line with the earlier studies presenting tube formation on basement membrane extracted from murine Engelbreth-Holm-Swarm (EHS) tumors^31^,^47^,^48^, where endothelial cells could form capillary-like structures even without additional stimuli^49^. A plausible explanation for the lack of capillary-like structures in the control group in the earlier study could be due to the fact that their primary HUVECs (bought from Invitrogen) were already differentiated and/or lost their endothelial phenotype in culture. We also found that tube formation along with the time course, at later time points was almost lost in the control and rhFSH-stimulated HUVECs. Only stimulation with the positive angiogenesis inducer rhVEGF significantly sustained their tube-like structures. The weaker response to rhVEGF stimulation in HUV-ST could be due to their immortalization process and perhaps to their higher passage numbers, as the original functional characteristics of this cell line showed formation of capillary-like structures in contact with Matrigel^48^.

Similarly to the above experiments, rhFSH stimulation did not affect the wound healing process in HUVEC and HUV-ST cells *vs*. control. Only rhVEGF stimulation as positive control closed the wounds within 36 h of incubation. The scratch/gap in wound healing assay performed in the earlier study was created manually using a 200 μL pipette tip (one well at a time)^15^. It has been shown that, making the scratch with a pipette tip may damage the extracellular matrix due to too much non-coordinated pressure, which can affect the migration rate^50^. To overcome this problem in our experiment, we used an automated device with multiple pins designed especially for wound healing assay^51^. Another important technical issue in the earlier study could be the rhFSH stimulation time of HUVECs^15^. They showed 20% (1/5 of analyzed area) of wounds were healed already after 4 h of rhFSHR stimulation. It is well known that wound healing requires both cell migration and proliferation in order to cover a gap made in confluent monolayer^22^. As the HUVEC cells presents with a doubling time for ~24 h, 4 h for this experimentation is too short a time for the cells to migrate and proliferate.

The lack of FSH-stimulated cAMP production and AKT phosphorylation additionally supported our negative results on functional FSHR expression in HUVEC and HUV-ST cells. The unexpected phosphorylated AKT (pAKT) in 4 h-starvated control as well as in rhFSH-stimulated cells in the previous study could be due to too short cell starvation time^15^. When we starved HUVECs for 8 h, no pAKT signal was observed in control and stimulated cells. This implies that 4 h of starvation in HUVECs might not be enough to suppress phosphorylation of AKT^52^,^53^ questioning the impact of rhFSH on HUVECs angiogenesis^15^.

Taken together, stimulation with rhFSH showed in our experiments no effect on proliferation, nitric oxide production, wound healing, tube formation assay, phosphorylation of AKT or cAMP production in HUVECs. Numerous methodological differences, resulting in hard-to-interpret findings in the previous study could explain the difference between these data^15^ and ours. Our results were not able to confirm the earlier finding of FSHR expression in HUVECs or in immortalized HUV-ST cells. Consequently, our data do not support the novel concept that FSH-FSHR activation is involved in the placental vasculature and their angiogenesis process. Thus, it remains open whether it is justified to conclude that FSH stimulation of the angiogenic process in HUVECs contributes to placental development and maintenance of healthy pregnancy^15^. Without FSHR expression in endothelial cells, FSH cannot be considered as a proangiogenic factor as VEGF. A weak FSHR signal in the nonpregnant human myometrium, which becomes upregulated in pregnant non-laboring human myometrium, as well as in muscle fibers, in the myometrial vessels has been shown earlier^8^. We were not able to detect FSHR signal in human myometrium^54^, both at mRNA or protein levels and on the contrary to the earlier sudy we sequenced our positive controls PCR products to reconfirm the PCR findings. Thus the recent study showing novel FSHR signaling pathway regulating myometrial contractibility in pregnancy^55^, is also in need of further confirmation. In light of our findings, more studies are definitely needed to validate the recently discovered extragonadal FSHR expression and function, especially in the endothelial cells of different vascular vessel types in normal and tumor tissues.

## Methods

### Cell culture and tissues

Human umbilical cords (UC) (n = 5 patients) were collected after a “written informed consent” was obtained from all study subject participants with healthy full-term and naturally delivered newborns at the Medical University of Bialystok (Poland). UCs were transported in cold phosphate-buffered saline (PBS) with 100 IU/mL penicillin and 100 mg/mL streptomycin (P/S solution; Sigma-Aldrich, St Louis, MO) to the laboratory. HUVECs were isolated from UC vein incubated with 0.1% collagenase in DMEM/F12 medium (Sigma-Aldrich) for 10 min at 37 °C as described previously^56^. Immunocytochemistry with antibodies directed against von Willebrand Factor (ab6994, Abcam, Cambridge, UK) and CD31 (ab28364, Abcam) were used to determine the endothelial cell purity. HUVECs were cultured in EGM2 (CC-3156 Lonza, Basel, Switzerland) medium supplemented with BulletKit (CC-4176 Lonza). HUV-ST cells (SV40Tag/telomerase-immortalized human umbilical vein endothelial cell line)^48^ were cultured in DMEM/F12 medium (Gibco, Thermo Fisher Scientific, Waltham, MA) with 10% fetal calf serum (FCS, Lonza). Both cell lines were incubated at 37 °C in a humidified atmosphere in the presence of 5% CO_2._ HUVEC (passages 2–4) and/or HUV-ST cells (passage 3–4) were used for cell stimulation studies. Granulosa cells (n = 4 donors) were obtained by follicular fluid aspiration from women undergoing *in vitro* fertilization treatment. After oocyte removal, follicular fluid containing granulosa cells were pooled and centrifuged at 250× *g* for 10 min. The fraction with granulosa cells was carefully aspirated, transferred to a new centrifuge tube, resuspended in PBS (Gibco) and centrifuged at 250× *g* for 5 min. Granulosa cell pellet was resuspended in DMEM/F12 medium (Sigma-Aldrich) with 10% FCS (Lonza) and cultured at 37 °C in a humidified atmosphere in the presence of 5% CO_2_. Archival granulosa cell tumor paraffin blocks (n = 5) were obtained from the Department of Pathology, Medical University of Bialystok. The local human Investigation Ethics Committee at the Medical University of Bialystok approved this study. All methods in this study were performed in accordance with the relevant guidelines and regulations.

### Hormones and growth factors

Human recombinant FSH (rhFSH) was purchased from National Hormone and Pituitary Program of NIDDK/NIH and received as a donation from Ferring Pharmaceuticals (Saint Prex, Switzerland). We did all the rhFSH stimulations with hormones from both sources and as the results were identical, we represented their mean values. Recombinant human VEGF 165 (rhVEGF, 293-VE/CF) and recombinant human FGF basic (rhFGF, 233-FB/CF) were purchased from R&D Systems (Minneapolis, MN). As Stilley *et al*. found^15^ rhFSH at a concentration of 600 ng/mL (equals to 8185.54 IU/L) causing the proangiogenic effect, we included the same concentration in our study. The other rhFSH concentrations tested were 0.73, 7.33 and 73.3 ng/mL, i.e. 10, 100 and 1000 IU/L of rhFSH, respectively.

### NO production

Prior to experiment cells were seeded onto 24-well plate (80000 cells/well). After overnight incubation in culture medium cell were starved for 4 h in basal non-supplemented EGM2 medium or for 1 h in Hank’s Balanced Salt Solution (HBSS). Then the cells were treated without (control) or with 0.733, 7.33, 73.3, and 600 ng/ml of rhFSH. Estradiol (E2, 10 ng/ml, Sigma) was used as a positive control for NO production^21^. 1 mmol/l of L-Nω-Nitroarginine Methyl Ester (L-NAME, Sigma) was used to inhibit constitutive NO synthase. Cells starved in basal culture medium were incubated with stimulants for 4 h whereas starved in HBSS for 1 h. Culture media and HBSS were collected and stored at −80 °C until further analysis.

In order to assess NO production total nitrite (NO_2_^−^) concentration was measured using Total Nitric Oxide and Nitrate/Nitrite Parameter Assay Kit (SKGE001, R&D Systems) with assay range 3.12 μmol/L–200 μmol/L. In a day of assay samples were thaw at RT. All reagents and standards’ serial dilutions were prepared according to nitrate reduction assay procedure. 50 μl of standards and samples were mixed with 25 μl of reduced β-nicotinamide adenine dinucleotide (NADH) and 25 μl of diluted nitrate reductase and incubated for 30 min at 37 °C. Then 50 μl of Griess Reagent I and 50 μl of Griess Reagent II were added to each well, mixed and incubated for 10 min at RT. Optical density was obtained using WALLAC Victor 2 1420 Spectrophotometer (Perkin Elmer) at 540 nm with wavelength correction at 690 nm.

### Tube formation assay

HUVEC and HU-ST cells upon reaching ~80% confluency were starved for 4 h in basal non-supplemented EGM2 medium and then seeded onto 96-well plate (15000 cells/well) covered with Matrigel (Geltrex™ LDEV-Free Reduced Growth Factor Basement Membrane Matrix, ThermoFisher Scientific) and treated without (control) or with 0.733, 7.33, 73.3, 600 ng/ml of rhFHS and rhVEGF (50 ng/ml) used as a positive control. In this assay, in the presence of angiogenic stimuli (rhVEGF and potentially rhFSH), the cells will form tube like structures. Specimen pictures were taken with converted light microscope (EVOS, Thermo Fisher Scientific) after 4, 8, 16, 24 and 32 h after seeding. Tube formation quantification was done using Angiogenesis Analyze for ImageJ software (Gilles Carpentier, Paris, France). Results were selectively confirmed manually.

### Wound healing assay

To investigate cell migration, we used the wound healing assay. HUVEC and HU-ST cells were seeded onto 96-well plate (10000 cells/well) and incubated in culture medium until reached a confluent monolayer. Then the cells were starved in basal non-supplemented EGM2 medium for 4 h. Scratch wound was created by automated 96-pin wound making tool (WoundMaker™; Essen Bioscience, Hertfordshire, UK). Cells were treated with: 0.733; 7.33; 73.3; 600 ng/ml of rhFSH and 50 ng/ml of rhVEGF used as a positive control in IncuCyte ZOOM^®^ (Essen Bioscience) for 36 h. Pictures were taken automatically every hour from two different places in the same well. Relative wound density was calculated by IncuCyte™ Chemotaxis Cell Migration Software (Essen Bioscience).

### AKT pathway/Western blotting

To analyse FSH-induced AKT pathway activation HUVEC and HU-ST cells were seeded onto 6-well plate (150000 cells/well), incubated overnight in culture medium and starved in basal non-supplemented EGM2 medium for 4 h and 8 h. Cells were treated with rhFSH (0.733, 7.33, 73.3 and 600 ng/ml) for 15, 30 and 60 min and scraped on ice with RIPA buffer (Thermo Fisher Scientific, Waltham, MA) with addition of protease and phosphatase inhibitors (cOmplete ULTRA Tablets and PhosSTOP Phosphatase Inhibitor Cocktail Tablets, Roche, Basel, Switzerland). Total protein concentration was measured with Bradford Protein Assay (Bio-Rad). Equal amounts of total proteins (25 μg) were separated on 10% polyacrylamide gels (1.5 h, 100 V, 4 °C). Gels were equilibrated with Towbin transfer buffer (25 mM Tris, 192 mM glycine, 20% methanol) for 20 min with gentle agitation. Proteins were transferred into polyvinylidene difluoride (PVDF) membranes (wet transfer, 2 h, 70 V). Before overnight incubation (4 °C with gentle agitation) with primary antibody either anti- phospho- AKT (Ser473, 4060, Cell Signalling Technology (CST) Danvers Massachusetts, USA) or anti-total AKT (9272, CST) membranes were blocked in 5% non-fat dry milk or in 5% BSA, respectively. Membranes were washed with TBS with 0.05% Tween (TBST) 3 × 5 min and incubated with anti-rabbit HRP-linked secondary antibody (CST) for 30 min at RT with gentle agitation. Membranes were washed 3 × 5 min with TBST and the Amersham Biosciences ECL detection system (GE Healthcare, Little Chalfont, UK) was used for protein visualization. Pictures were taken using ImageQuant LAS 4000 (GE Healthcare).

### Statistical analysis

Data are presented as mean ± S.E.M. To assess statistical significance in proliferation assay, NO and cAMP production, one-way ANOVA with Dunnett’s multiple comparison test with 95% confidence interval was used (GraphPad PRISM v5.0, GraphPad Software Inc., San Diego, CA). Two-way ANOVA with Bonferroni comparison with 95% confidence interval was used to test dose-, and time-dependent tube formation and cell migration (GraphPad PRISM v. 5). Results were considered significant at P < 0.05 level and are denoted by an asterisk (*).

### Total RNA isolation, reverse transcription and quantitative PCR; RNAscope^®^ *in situ* hybridization; immunocytochemistry, Proliferation assay and cAMP production

These methods are provided in Supplementary Materials and Methods.

## Additional Information

**How to cite this article**: Stelmaszewska, J. *et al*. Revisiting the expression and function of follicle-stimulation hormone receptor in human umbilical vein endothelial cells. *Sci. Rep.* **6**, 37095; doi: 10.1038/srep37095 (2016).

**Publisher's note**: Springer Nature remains neutral with regard to jurisdictional claims in published maps and institutional affiliations.

## Supplementary Material

Supplementary Information
