# Supplementary material for: Revisiting the expression and function of follicle-stimulation hormone receptor in human umbilical vein endothelial cells
Source: Sci Rep. 2016 Nov 16;6:37095. doi: 10.1038/srep37095 (PMC5111068; doi:10.1038/srep37095)

**Supplementary Appendix**

**Supplement to:**

**Revisiting the expression and function of follicle-stimulation hormone receptor in human umbilical vein endothelial cells**

Joanna Stelmaszewska, Marcin Chrusciel, Milena Doroszko, Malin Akerfelt, Donata Ponikwicka-Tyszko, Matthias Nees, Marco Frentsch, Xiangdong Li, Jukka Kero, Ilpo Huhtaniemi, Slawomir Wolczynski, Nafis A. Rahman

**Table of contents**

Supplementary Materials and Methods sp. 3

Supplementary Figures/Table/References/full-length blots & gels

Figure S1 sp. 7

Figure S2 sp. 7

Figure S3 sp. 8

Figure S4 sp. 9

Table S1 sp.10

Supplementary References sp. 10

Supplementary full-length blots/gels sp.11

**Supplementary Materials and Methods.**

**Total RNA isolation, reverse transcription and quantitative PCR**

Fragments of umbilical cord vein and arteries were dissected from the umbilical cord immediately after arrival to the laboratory. The whole procedure was performed on sterile Petri dish placed on ice. Fragments of UC were snap frozen in liquid nitrogen and stored at -80 °C. Total RNA from umbilical cord and cell lines was isolated using the Trizol-based standard extraction protocol (Life Technologies, Thermo Fisher Scientific). Total RNA concentration was quantified using a NanoDrop 1000 spectrophotometer (NanoDrop Technologies, Wilmington, DL). Revers transcription was performed with two kits: SensiFAST™ cDNA Synthesis Kit (Bioline, London, UK) and Maxima First Strand DNA Synthesis Kit (Thermo Fisher Scientific). Prior cDNA synthesis with SensiFAST™ cDNA Synthesis Kit 1 μg of total RNA was incubated with 1 μL of DNase I (amplification grade, 1 U/μl) and 1 μL of 10X reaction buffer (DNase I Amplification Grade, Sigma-Aldrich) at room temperature (RT) for 15 min Next, 1 μL of stop solution was added, incubated at 70 °C to inactivate the enzyme for 10 min, and chilled on ice. DNase I-treated RNA (11 μL) was mixed with 4 μL of 5X TransAmp buffer, 1 μL of reverse transcriptase and 4 μL of nuclease free water (NFW). Reaction was carried in thermal cycler in following conditions: 25 °C for 10 min, 42 °C for 15 min, 48 °C for 15 min (additional step for highly-structured RNA), 85 °C for 5 min, 4 °C to hold. For reverse transcription with Maxima First Strand DNA Synthesis Kit 1 μL of 10X dsDNase buffer and 1 μL of dsDNase were used with 1 μg of RNA, mixed, centrifuged, incubated 2 min at 37 °C and chilled on ice. Next 4 μl of 5X reaction mix, 2 μL of maxima enzyme mix and 4 μL of NFW were added. Conditions for reverse transcription were as follows: 25 °C for 10 min, 65 °C for 15 min, 85 °C for 5 min, 4 °C to hold.

Real time quantitative polymerase chain reactions (qPCR) were performed with DyNAmo HS SYBR Green qPCR Kit (Thermo Fisher Scientific) using standard primers (Supplementary Table 1) and/or TaqMan® Fast Advanced Master Mix with TaqMan probes (Applied Biosystems, Thermo Fisher Scientific) (Supplementary Table 1). qPCR was carried in CFX96 Touch™ Real-Time PCR Detection System (Bio-Rad, Hercules, CA). For SYBR-based qPCR 15 ng of cDNA in 8 μL, 10 μL of DyNAmo HS SYBR Green Master Mix and 2 μL of 1 μM forward and reverse primer were used (total volume 20 μl) and run under following conditions: 2 min at 50 °C, 10 min at 95 °C, 40 cycles of 15 s at 95 °C and 1 min at temperature specific for primer (Table 1). For TaqMan-based qPCR 15 ng of template diluted in 8 μl NFW was mixed with 10 μL of TaqMan® Fast Advanced Master Mix and 1 μL of 1X TaqMan probe and 1μL of NFW (total volume 20 μL) and run with the following conditions: 2 min at 50 °C, 20 min at 95 °C, 40 cycles of 1 s at 95 °C and 20 s at 60 °C. Each and every reaction products were separated and verified by sequencing analysis.

**RNAscope® in situ hybridization**

*In situ* hybridization of formalin fixed paraffin embedded (FFPE) blocks of umbilical cord vein and human granulosa cell tumor were done with RNAscope FFPE 2.0 HD Detection Kit Brown (1) [Advanced Cell Diagnostics (ACD), Hayward, CA, CAT# 310033]. FFPE blocks of UC tissues, human granulosa cell tumors (as positive controls for FSHR) were cut in 5 μm sections, baked 1 h at 60 °C and deparaffinised within one week after cutting (2 x 5 min in fresh xylene, 2 x 1 min in 100% ethanol). After drying for 5 min at RT pretreat 1 was applied on the sections and incubated for 10 min at RT. Than slides were washed twice with deionized water (ddH_2_O) and boiled in pretreat 2 for 15 min. Slides were immediately washed twice with ddH_2_O and 100% ethanol and air-dried overnight at RT. The next day pretreat 3 was applied for 30 min at 40 °C in HybEZ(TM) Oven (ACD). Next slides were washed twice with ddH_2_O. Pre-warmed probes: *FSHR* probe (Hs-FSHR-ver2, ACD-408101), positive control probe for low abundance transcripts (*HS-POLR2A*, ACD-310451) and negative control probe (*DapB,* ACD-310043), were added on the sections for 2 h at 40 °C in HybEZ(TM) Oven (ACT). Slides were washed twice for 2 min and incubated with subsequent hybridization amplifiers (AMPs) for 30 min (AMP 1, 3, 5) or 15 min (AMP 2, 4, 6) at 40 °C in HybEZ(TM) Oven with double washes (1X wash buffer for 2 min) in between. Next equal volume mixture of brown-A and brown-B was added and sections were incubated at RT for 10 min. After double washing with ddH_2_O and counterstaining 2 min with fresh 50% Gill's Hematoxylin (Vector Laboratories, Burlingame, CA) slides were washed with ddH_2_O and incubated in 0.02% ammonia water until sections turned blue. Dehydration was done with fresh ethanol (70% for 2 min, twice with 100% for 2 min) and xylene for 5 min. Slides were mounted with Pertex (Histolab Products AB, Gothenburg, Sweden).

**Immunocytochemistry**

Cells were grown on Millicell EZ Slide 8-well glass (Merck Millipore, Darmstadt, Germany) overnight in culture medium, fixed with 4% paraformaldehyde in PBS (15 min, RT) and washed with PBS (3 x 5 min). In order to permeabilize cell membranes 01% Triton X-100 in PBS was used (10 min, RT). Cells were washed in PBS (3 x 5 min) and incubated 30 min, with blocking solution (3% BSA in PBS with 0.05% Tween20; PBST) at RT. Thereafter cells were incubated in humidified chamber for 1 h at RT with following antibodies anti: von Willebrand Factor (ab6994, Abcam, Cambridge, UK), CD31 (ab28364, Abcam), FSHR323 (5μg/ml; “in-house” antibody kindly donated by Dr Ghinea), FOXL2 (ab5096,1:100, Abcam) and FLAG (F7425,1:300, Sigma-Aldrich) diluted in blocking solution. Next slides were washed with PBS (3 x 5 min) and incubated with goat anti-rabbit, goat anti-mouse or donkey anti-goat IgG conjugated with Alexa Fluor 488 or Alexa Fluor 594 (all diluted 1:250, Thermo Fisher Scientific) for 45 min in the dark (RT). After washing with PBS (3 x 5 min) cell nuclei were stained with DAPI (1 μg/ml in PBS) for 5 min at RT, washed 3 times with PBS and mounted in mounting medium (101098-042, Vector Laboratories, Burlingame, California, USA).

**Proliferation assay**

Proliferation of HUVECs and HUV-ST cells was determined using CyQUANT® Cell Proliferation Assay (C7026, Life Technologies, Thermo Fisher Scientific). Prior to experiment HUVEC and HUV-ST cells were seeded onto 96-well plate (5000 cells/well). After overnight incubation in culture medium cells were starved for 4 h in basal non-supplemented EGM2 medium. Then, cells were treated for 24 h with rhFSH in following concentrations: without (control) or with 0.733 (10 IU/L); 7.33 (100 IU/L); 73.3 (1000 IU/L); 600 ng/ml (8185.54 IU/L), and rhVEGF (50 ng/ml) as positive control. After medium removal, cells were washed with PBS and frozen at -80 °C. Next day plate was thaw at RT and 200 μl of the CyQUANT® GR dye/cell-lysis buffer (prepared according to manufactures instructions) was added and incubated for 4 min at RT, protected from light. For DNA standard curve serial dilutions of bacteriophage λ DNA (ranging from 50 pg/ml to 1.0 μg/ml) were done using CyQUANT® GR/cell-lysis buffer. Moreover control without DNA was also included. Fluorescence was read using WALLAC Victor 2 1420 Spectrophotometer (Perkin Elmer, Waltham, Massachusetts, USA) with ~480 nm excitation and ~520 nm emission maxima.

**cAMP production**

In order to measure FSH-stimulated cAMP production, HUVEC and HUV-ST cells were seeded onto 24-well plates (80000 cells/ well) and grown overnight in culture medium. Before the stimulation without (control) or with 0.733, 7.33, 73.3 and 600 ng/ml of rhFSH or 10 μM forskolin (FRK, used as a positive control), cells were starved for 4 h. cAMP production was enhanced with 3-isobutyl-1-methylxanthine (IBMX; Sigma) a competitive non-selective phosphodiesterase inhibitor added to the stimulation medium. Cells were incubated for 1 h at 37 °C, in 5% CO_2_ humidified atmosphere, and then 300 μl of medium was mixed with equal amount of 2 mM theophylline (Sigma) to protect cAMP degradation. Tubes were boiled 3 min in water bath, chilled on ice and kept in -80 °C for further analysis.

Extracellular cAMP production was determined according to the standard radioimmunoassay method described by Harper and Brooker (2) using iodinated succinyl-cAMP. Before the assay ^125^I-cAMP stock was diluted with 0.1% BSA/PBS solution to reach 14000 cpm/100 μl (cpm-counts per minute). Next, 300 μl of each sample, 0.1% BSA/PBS solution (for blank and reference) and cAMP standards (serial dilution range 10000-0-9.75 fmol/100 μl) were transferred to a new glass tube and acetylated with freshly prepared 10 μl of triethylamine/acetic anhydride mix (2:1) (both Sigma). Then, 2x 100 μl from each glass tube were transferred into two new plastic tubes and incubated overnight at 4 °C as follows: 1) blank samples – with 100 μl 0.1% BSA/PBS and 100 μl of ^125^I-cAMP 2) reference, standard and cell culture samples - with 100 μl of ^125^I-cAMP and 100 μl of cAMP antiserum; 3) two total samples containing only 100 μl of ^125^I-cAMP were used as internal control of the assay. The next day to each sample (beside total) 200 μl of 0.5% globulin in PBS and 2 ml of 16% polyethylene glycol 6000 in PBS (both Sigma) were added, vortexed, incubated for 30 min at 4 °C and centrifuged at 3000 rpm for 20 min at 4 °C. After the supernatants were completely removed and the rest of the humidity from the tube wall was taken away, radiation of the pellets were measured by γ-counter and automatically recalculated to fmol concentration (Wallac 1470 Wizard Gamma Counter, Perkin Elmer).

**Supplementary Figures**


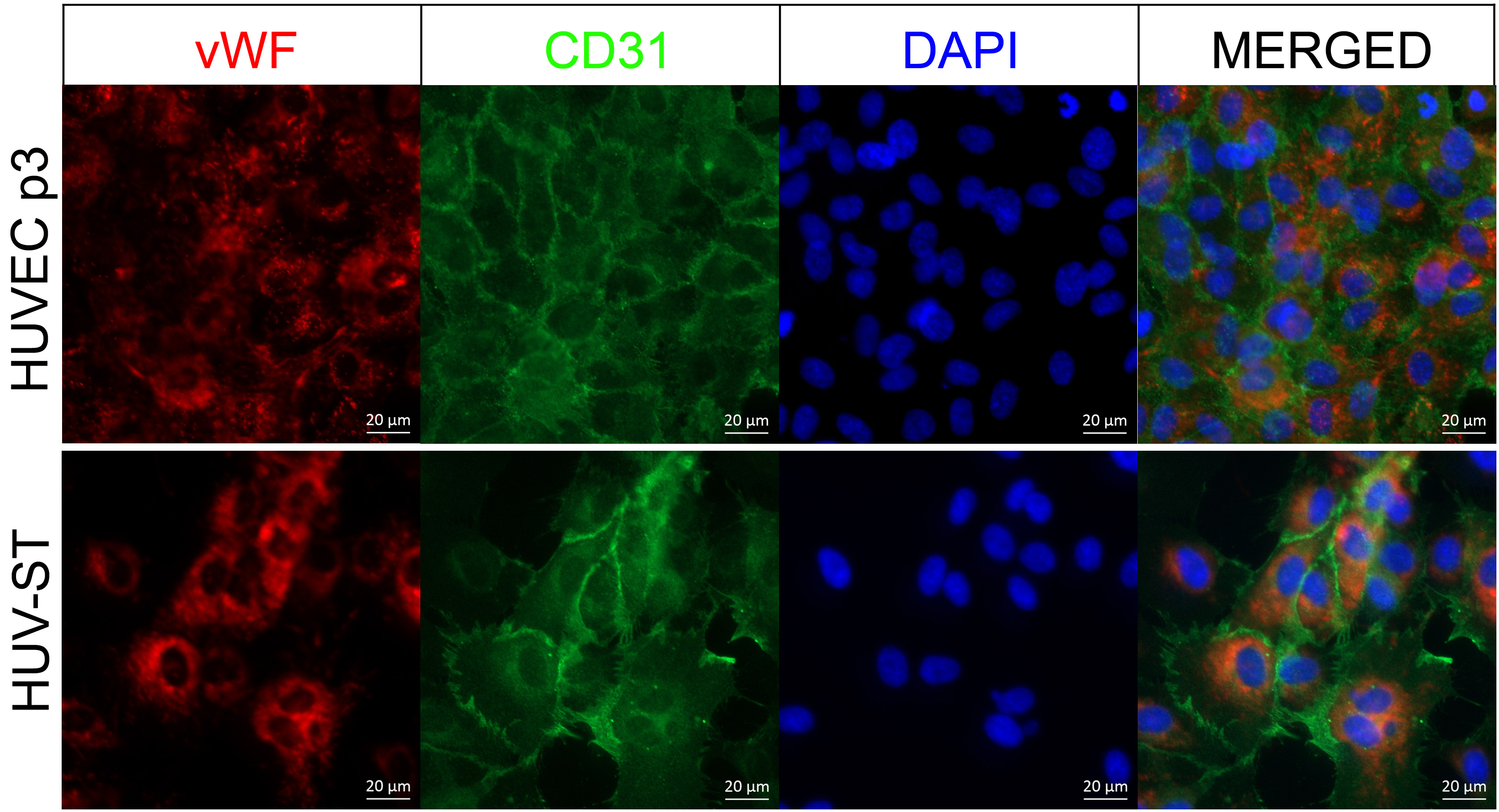


**Supplementary Figure 1**. Expression of endothelial cell markers: von Willebrand Factor (vWF, red) and CD31 (green) in HUVECs and HUV-ST cells. DAPI was used as counterstaining to detect cell nuclei (blue).


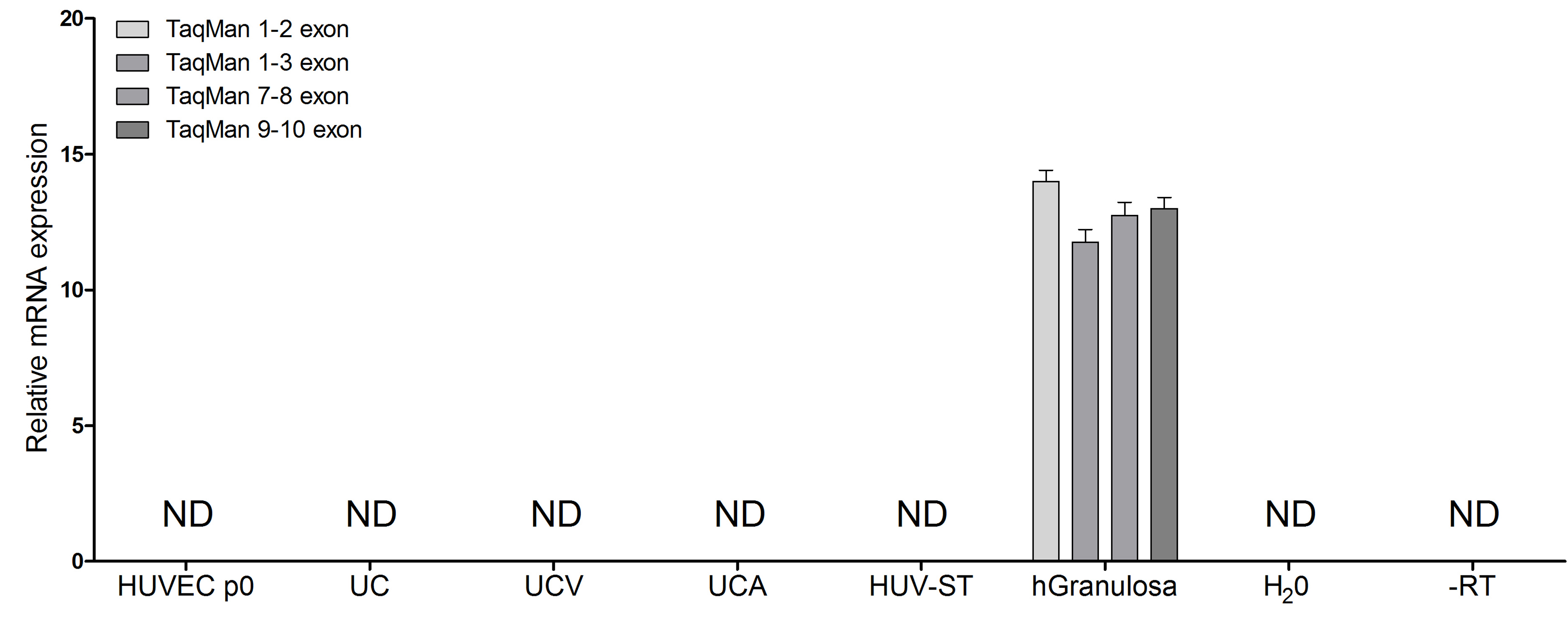


**Supplementary Figure 2.** Expression of *FSHR* transcripts analyzed by TaqMan probe-based qPCR. *FSHR* expression was analysed with TaqMan probes spanned different exons of *FSHR* using cDNA from HUVEC (passage 1or 0), umbilical cord (UC), umbilical cord vein (UCV), umbilical cord artery (UCA), HUV-ST cell line and human granulosa cells as a positive control. A no reverse transcriptase control (-RT) and no template control (H_2_O) were used as negative controls. Data are presented as relative expression to housekeeping gene human peptidylprolyl isomerase A (*PPIA*).


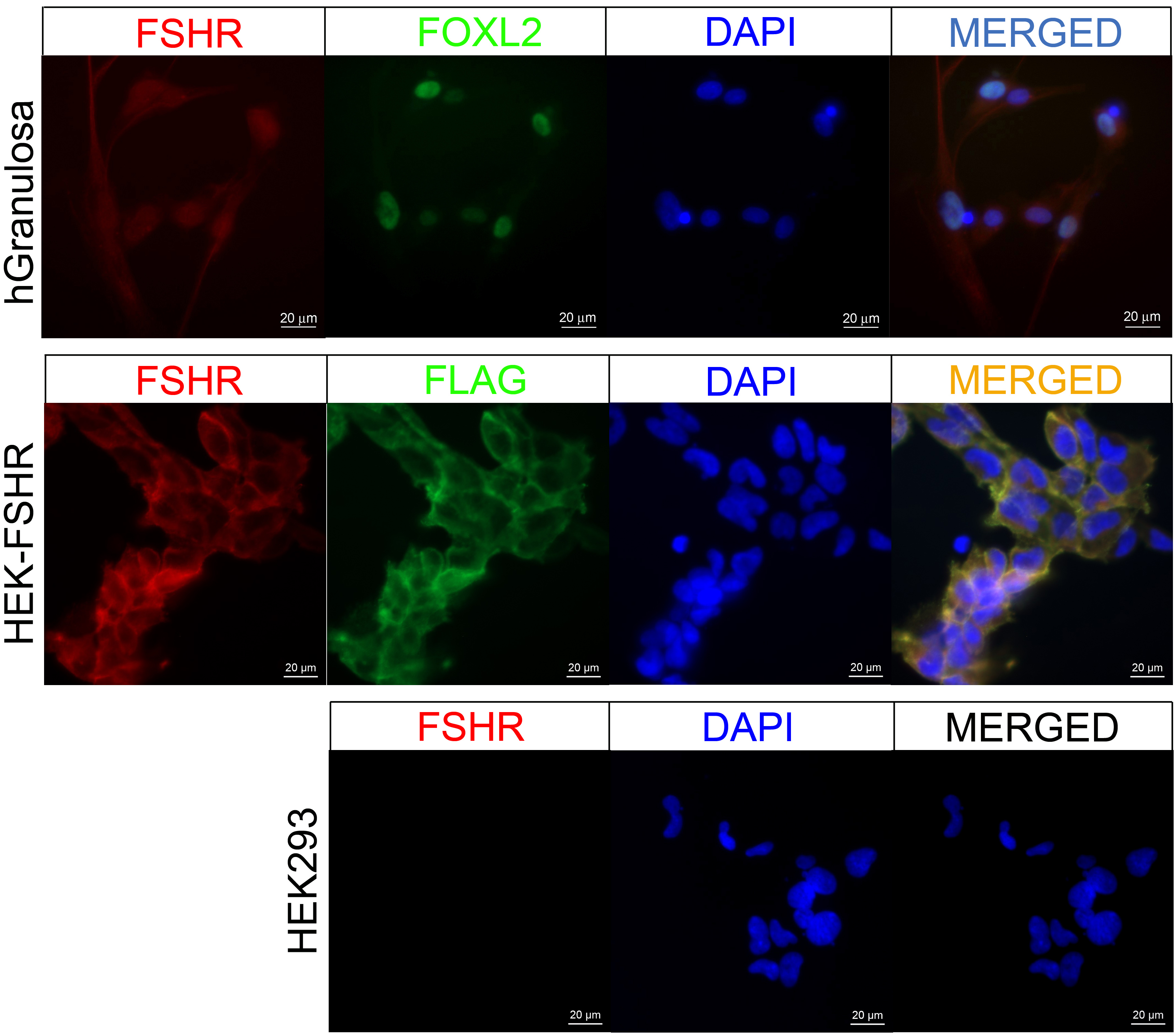


**Supplementary Figure 3**. Immunocytochemical localization of FSHR (red) in human granulosa, HEK-FSHR and HEK293 cell lines. FOXL2 (green) was used as a granulosa cell marker where FLAG peptide (green) was used as a reporter sequence to detect FSHR construct in stably transfected HEK293 cells (HEK-FSHR cells). DAPI was used as counterstaining to detect cell nuclei (blue).

**
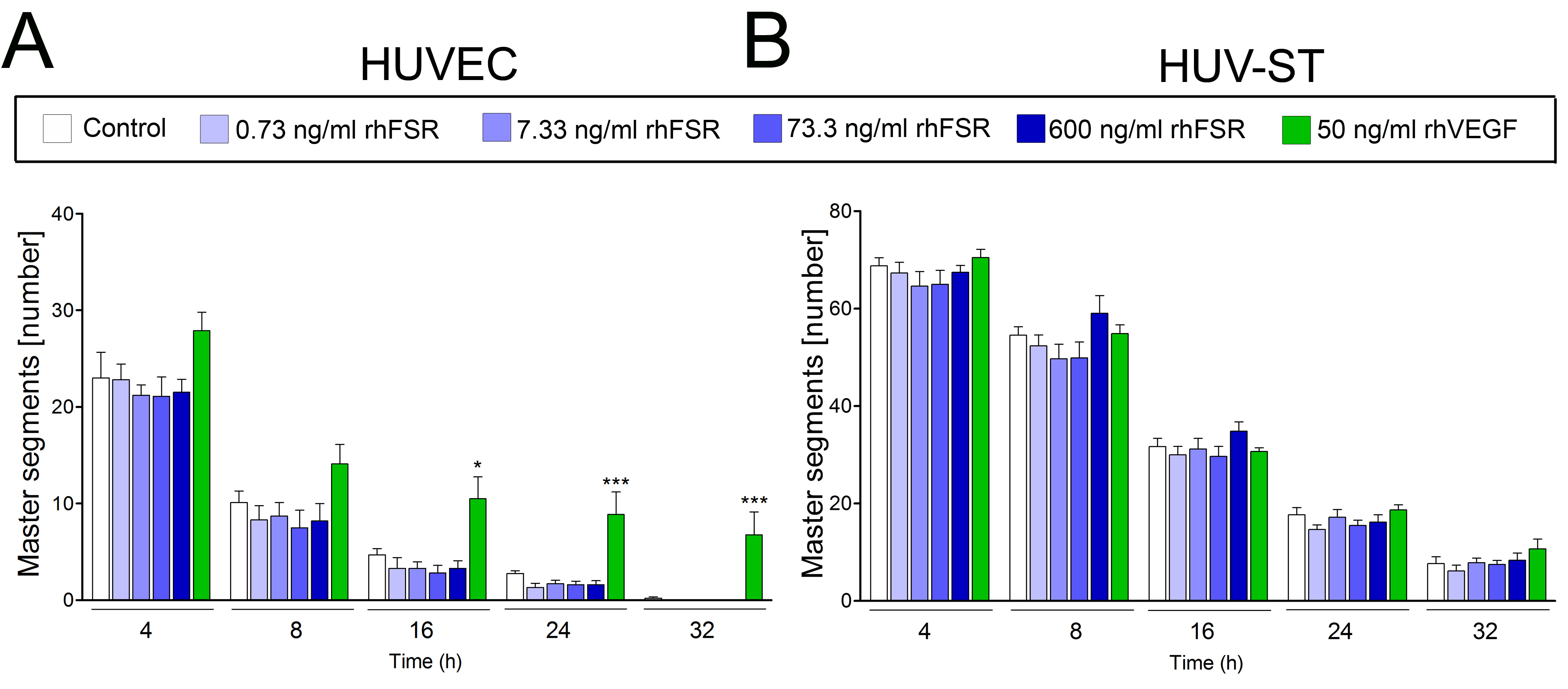
**

**Supplementary Figure 4**. Number of segments created by HUVEC (A) and HUV-ST (B) cells. Cells seeded on reduced growth factor basement membrane matrix were stimulated without or with 0.733, 7.33, 73.3 and 600 ng/ml of rhFSH, or 50 ng/ml rhVEGF used as a positive control. Pictures were taken after 4, 8, 16, 28 and 32 h and number of master segments was measured by Angiogenesis Analyzer for ImageJ software and selectively confirmed manually. Each bar represents the mean ± SEM of three independent experiments with n=5 per treatment. Asterisks indicate differences between control and stimulated cells (*P<0.05, **P<0.01, ***P<0.001).

Supplementary Table 1. Primer and TaqMan probe sequences and GenBank accession numbers for qPCR gene expression analyses.

| Gene/NCBI Reference | Spanning exons | Primers sequence (5’-3’) | Product size (bp) | Annealing temperature |
| --- | --- | --- | --- | --- |
| FSHR  NM_000145.3 | 1-2exons | F: GCCAAGAGAGCAAGGTGACA  R: CTCGAAGCTTGGTGAGGACA | 84 | 60 |
| FSHR  NM_000145.3 | 1-3exons | F: GGAATGCCATTGAACTGAGG  R: TTGGGAAGGTTGGAGAACAC | 151 | 58 |
| FSHR  NM_000145.3 | 7-8exons | F: ACAACTGTGCATTCAATGGAACC  R: GAATGACTGGTCCAGAGGCT | 111 | 60 |
| FSHR  NM_000145.3 | 9-10exons | F: GAAGCCAGCCTCACCTATCC  R: GGATCTCTGACCCCTAGCCT | 141 | 60 |
| FSHR  NM_000145.3 | Variant 1 | F: AGGCCAACAACCTGCTCTAC  R: CTTTCAAAGCTCAGCCCCAC | 205 | 60 |
| FSHR  NM_000145.3 | 1-2exons | F: GCCAAGAGAGCAAGGTGACA  R: CTCGAAGCTTGGTGAGGACA  PROBE: GACCTCCCGAGGAA | 84 | 60 |
| FSHR  NM_000145.3 | 1-3exons | F: GGAATGCCATTGAACTGAGG  R: TTGGGAAGGTTGGAGAACAC  PROBE: AGAGATCTCTCAGAATGA | 151 | 58 |
| FSHR  NM_000145.3 | 7-8exons | F: ACAACTGTGCATTCAATGGAACC  R: GAATGACTGGTCCAGAGGCT  PROBE: GAGCTGAATCTAAGCG | 111 | 60 |
| FSHR  NM_000145.3 | 9-10exons | F: GAAGCCAGCCTCACCTATCC  R: GGATCTCTGACCCCTAGCCT  PROBE: CTCTGAGCTTCATCCA | 141 | 60 |
| FSHR  NM_000145.3 | Variant 1 | F: AGGCCAACAACCTGCTCTAC  R: CTTTCAAAGCTCAGCCCCAC  PROBE: CCTTCCAGATGTTC | 205 | 60 |
| ACTB  NM_001101.3 | 2-3exon | F: ACTTCGAGCAAGAGATGGCCA  R: GACTCCATGCCCAGGAAGGA | 148 | 61 |

Supplementary References

**1.** Wang F, Flanagan J, Su N, Wang LC, Bui S, Nielson A, Wu X, Vo HT, Ma XJ, Luo Y. RNAscope: a novel in situ RNA analysis platform for formalin-fixed, paraffin-embedded tissues. The Journal of molecular diagnostics : JMD 2012; 14:22-29

**2.** Harper JF, Brooker G. Femtomole sensitive radioimmunoassay for cyclic AMP and cyclic GMP after 2'0 acetylation by acetic anhydride in aqueous solution. Journal of cyclic nucleotide research 1975; 1:207-218

Below, the full-length blots/gels of the cropped gel/band figures presented in the manuscript:

SUPPL. full length blots for Fig.1A


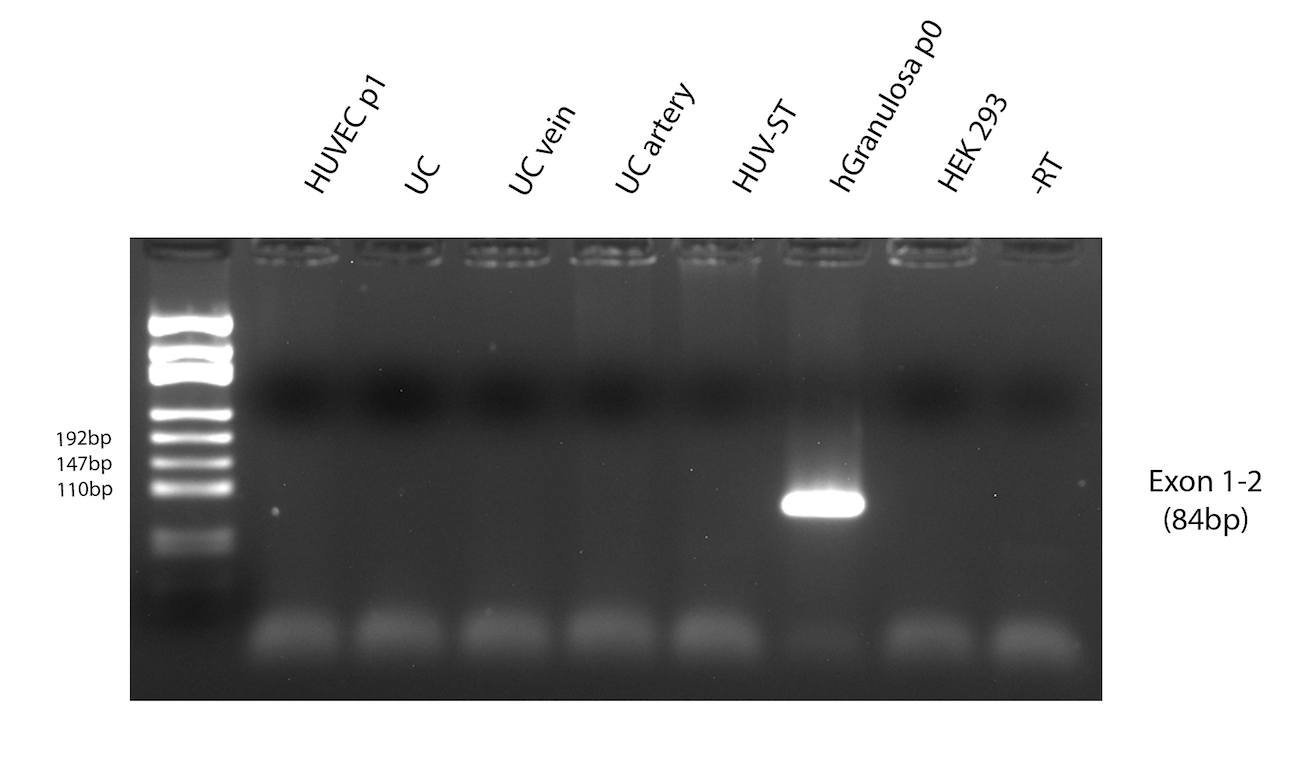


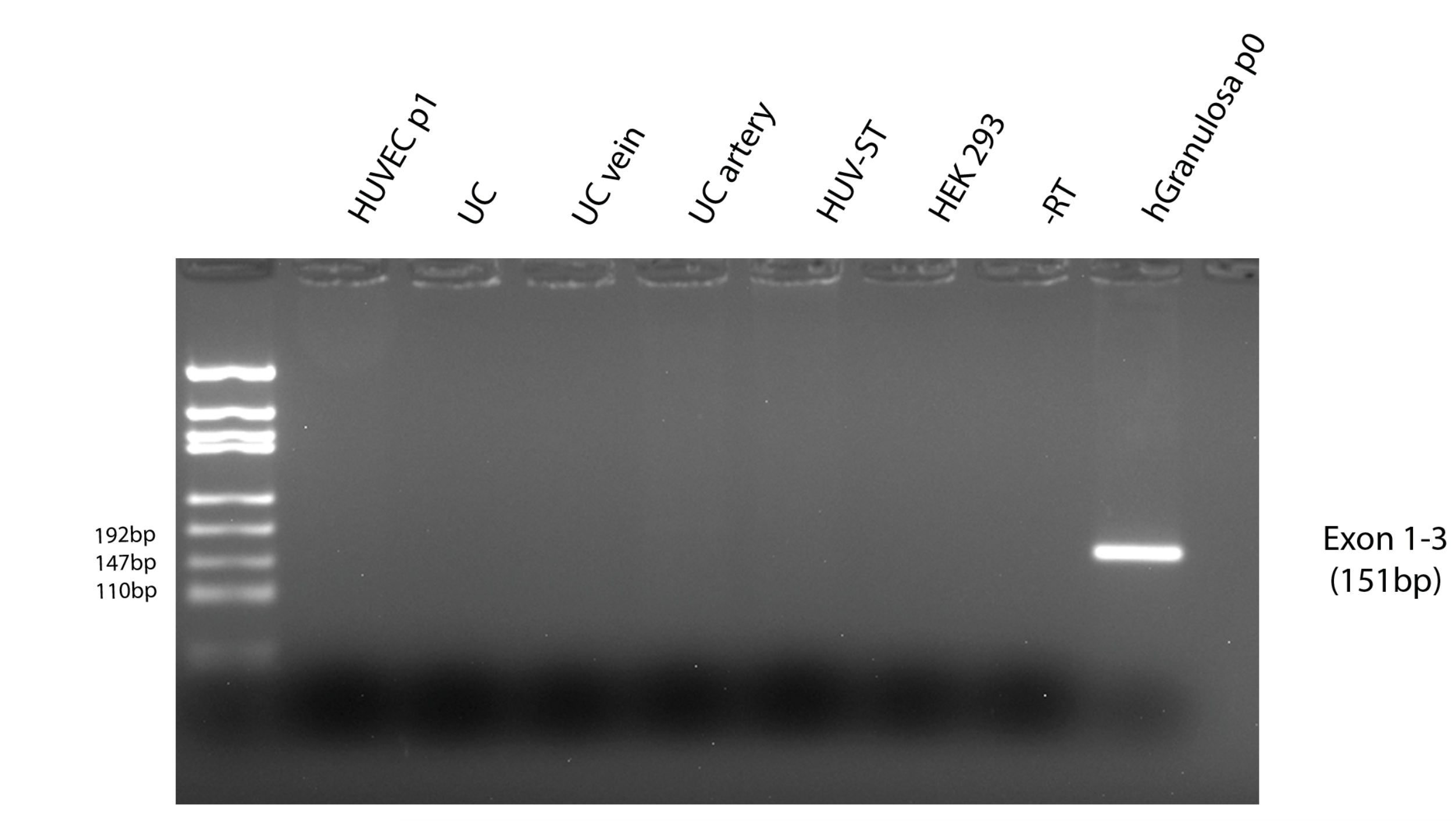


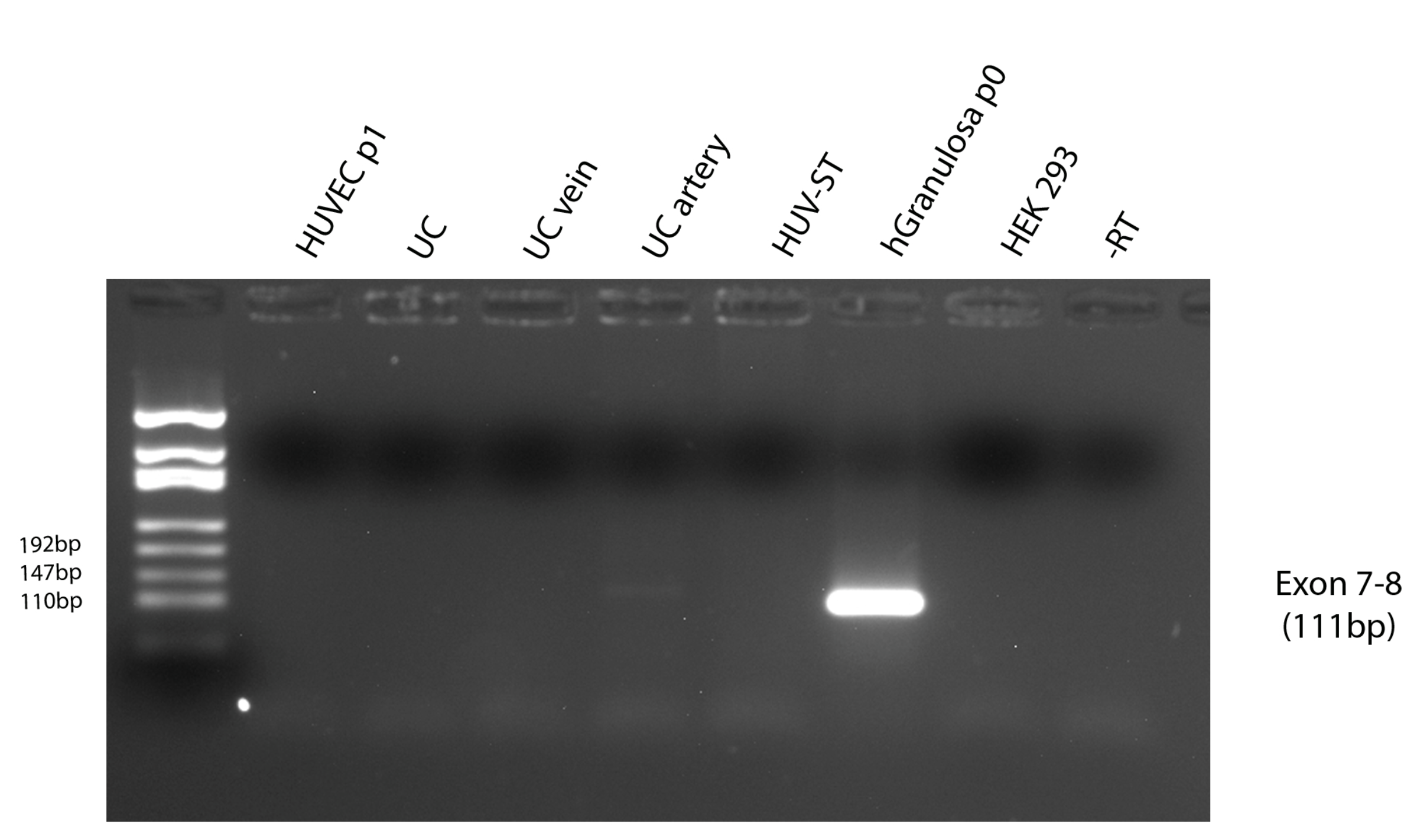


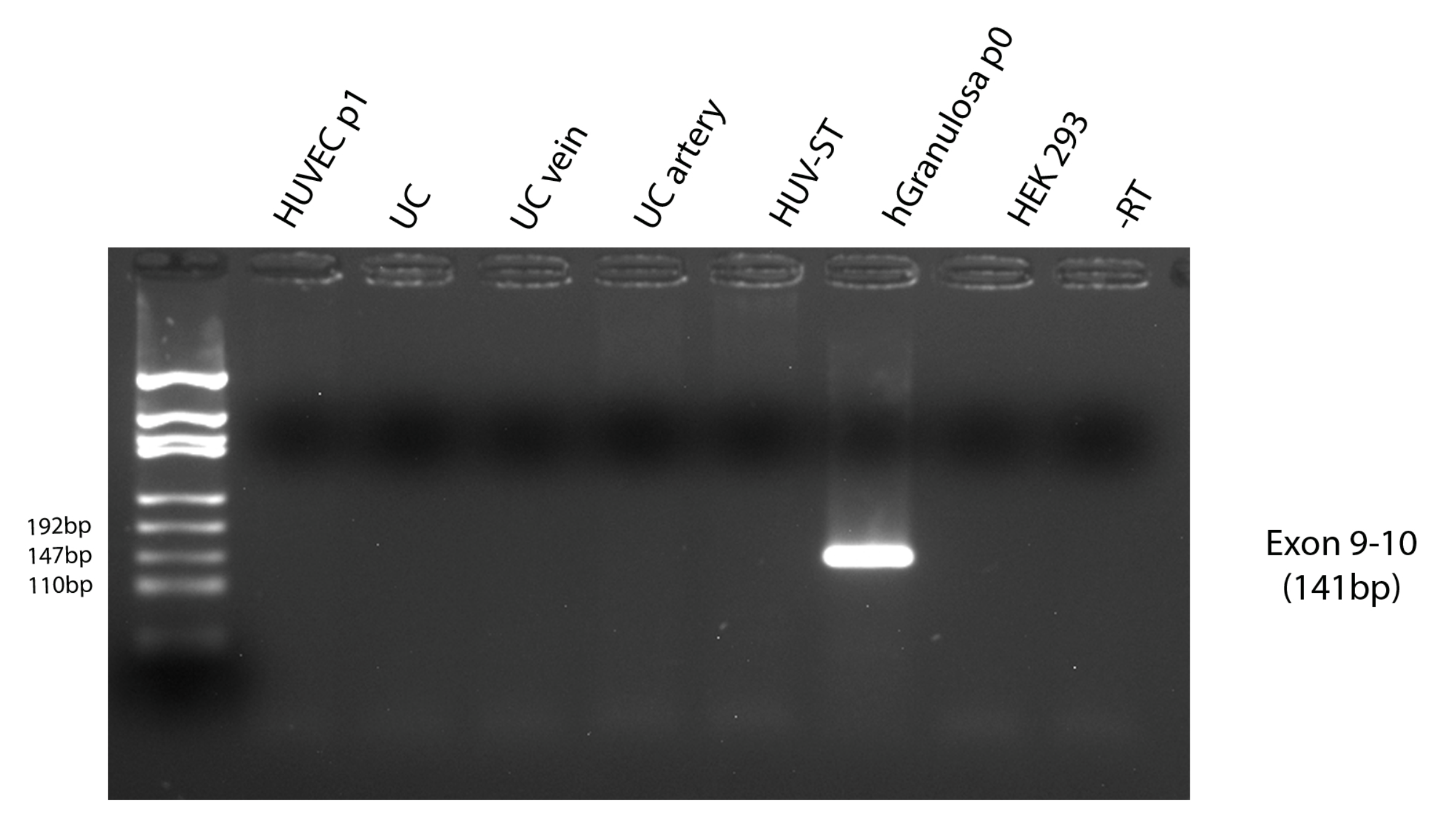


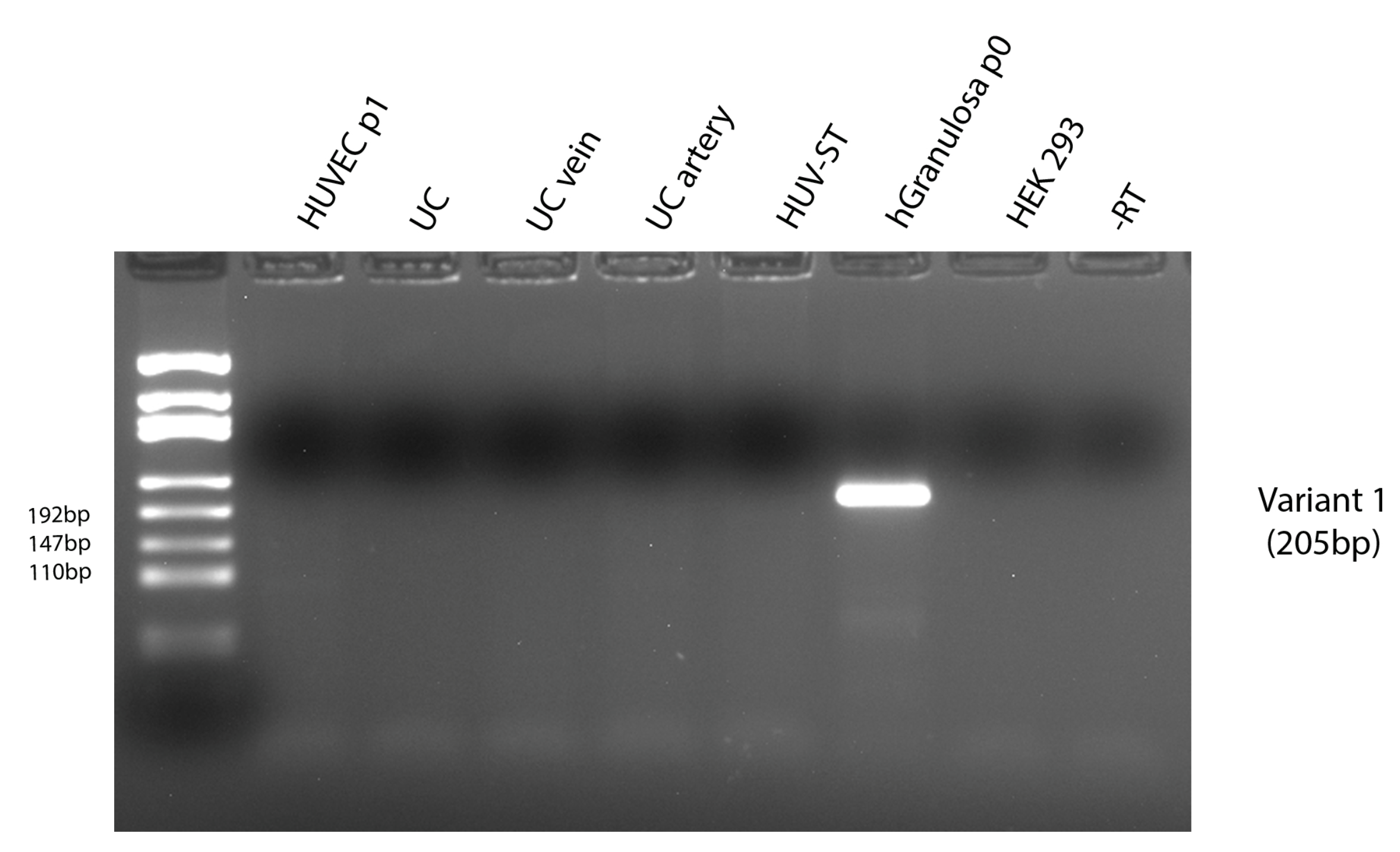


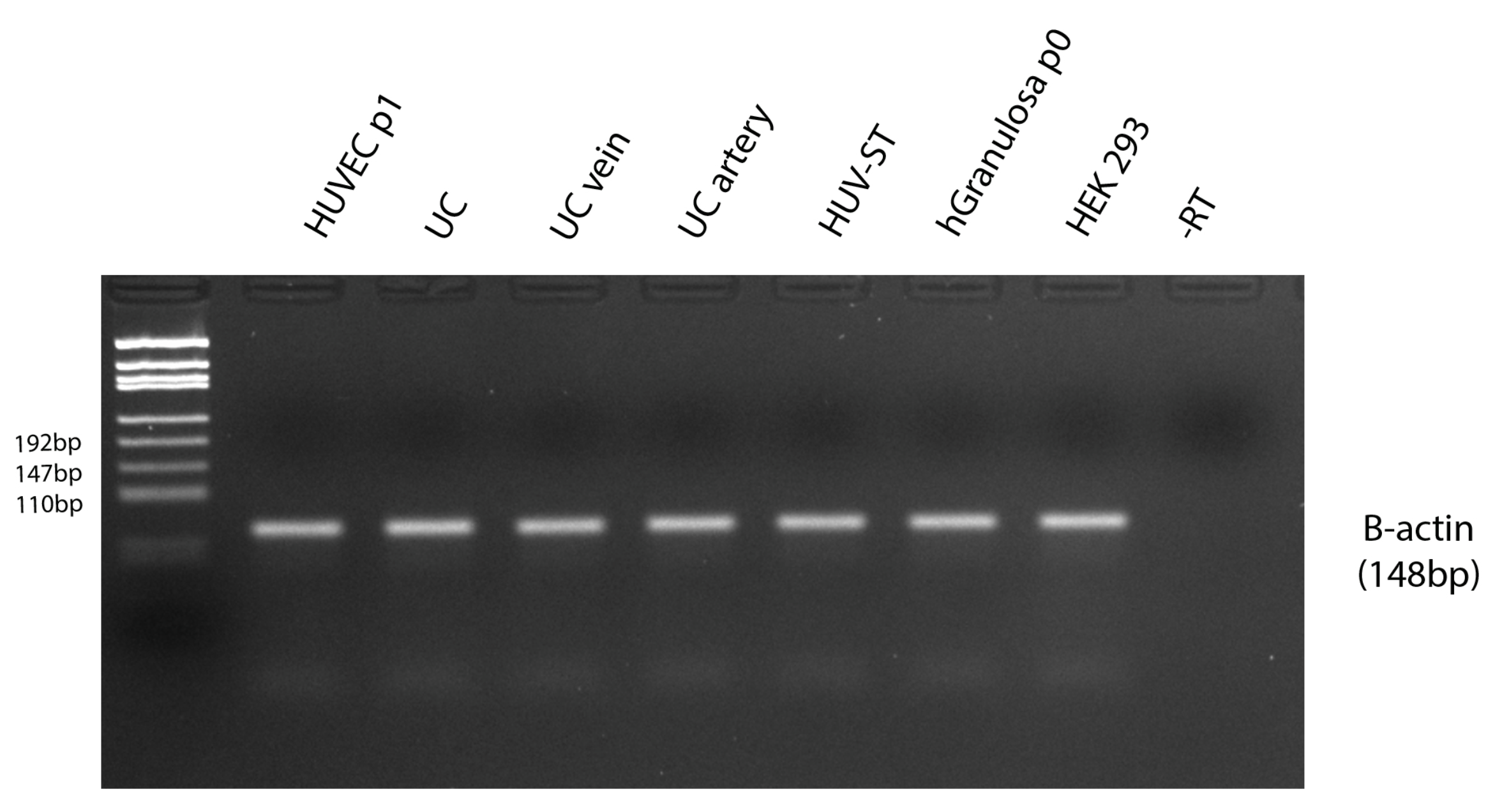


SUPPL. full length blots for Fig. 6B


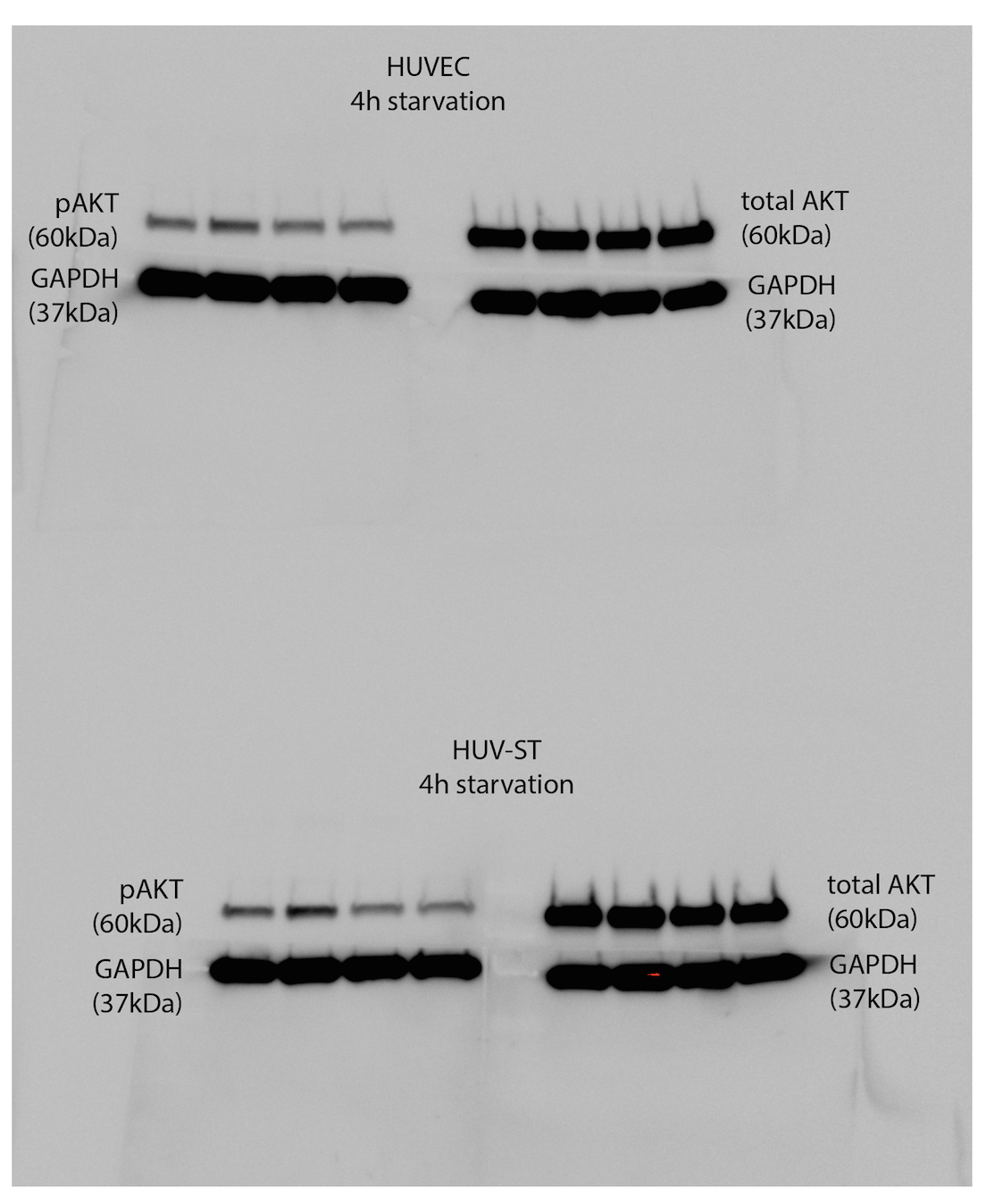


SUPPL. full length blots for Fig. 6C


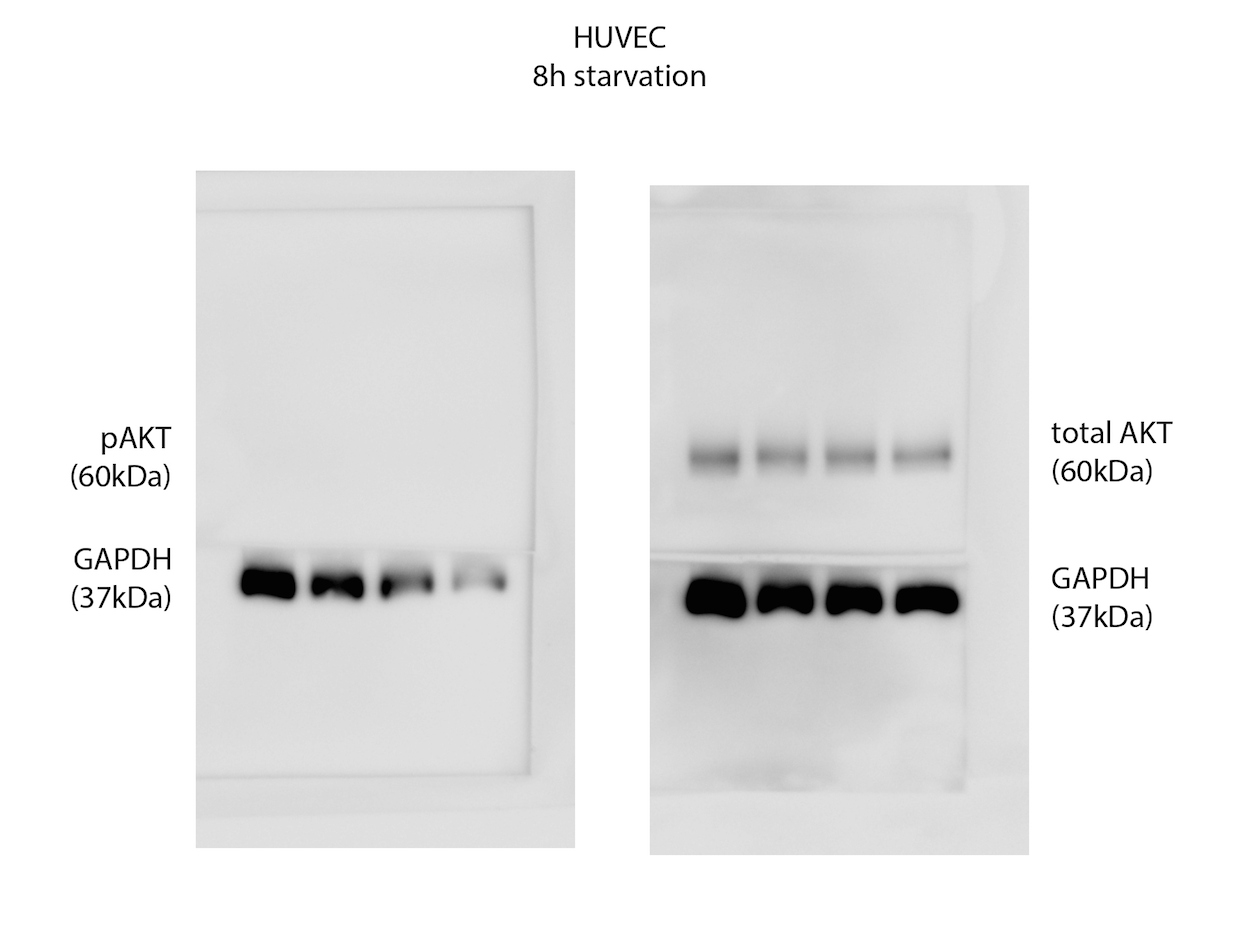


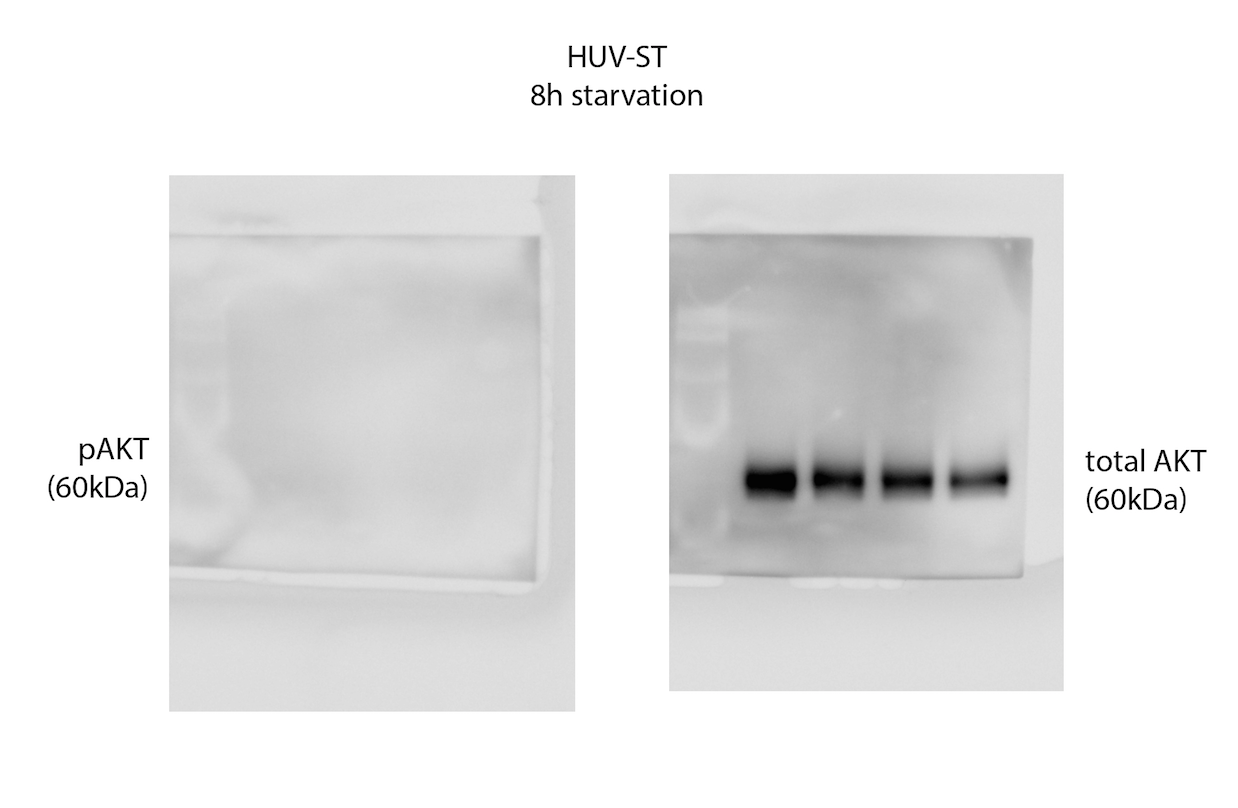

Supplement: Supplementary Information [file srep37095-s1.doc]
